# Dynamic Simulation Models of Suicide and Suicide-Related Behaviors: Systematic Review

**DOI:** 10.2196/63195

**Published:** 2024-12-02

**Authors:** Genevieve Gariepy, Rifat Zahan, Nathaniel D Osgood, Benjamin Yeoh, Eva Graham, Heather Orpana

**Affiliations:** 1 Centre for Surveillance and Applied Research Public Health Agency of Canada Ottawa, ON Canada; 2 Department of Computer Science University of Saskatchewan Saskatoon, SK Canada; 3 Department of Community Health & Epidemiology University of Saskatchewan Saskatoon, SK Canada; 4 Department of Epidemiology, Biostatistics, and Occupational Health Faculty of Medicine and Health Sciences McGill University Montreal, QC Canada; 5 Division of Epidemiology Dalla Lana School of Public Health University of Toronto Toronto, ON Canada; 6 School of Psychology University of Ottawa Ottawa, ON Canada

**Keywords:** suicide, agent-based modeling, complex system, complexity science, discrete-event simulation, dynamic modeling, microsimulation, system dynamics, systems science, qualitative study, dynamic simulation, database, depression, mental state, systematic review, stress

## Abstract

**Background:**

Suicide remains a public health priority worldwide with over 700,000 deaths annually, ranking as a leading cause of death among young adults. Traditional research methodologies have often fallen short in capturing the multifaceted nature of suicide, focusing on isolated risk factors rather than the complex interplay of individual, social, and environmental influences. Recognizing these limitations, there is a growing recognition of the value of dynamic simulation modeling to inform suicide prevention planning.

**Objective:**

This systematic review aims to provide a comprehensive overview of existing dynamic models of population-level suicide and suicide-related behaviors, and to summarize their methodologies, applications, and outcomes.

**Methods:**

Eight databases were searched, including MEDLINE, Embase, PsycINFO, Scopus, Compendex, ACM Digital Library, IEEE Xplore, and medRxiv, from inception to July 2023. We developed a search strategy in consultation with a research librarian. Two reviewers independently conducted the title and abstract and full-text screenings including studies using dynamic modeling methods (eg, System Dynamics and agent-based modeling) for suicide or suicide-related behaviors at the population level, and excluding studies on microbiology, bioinformatics, pharmacology, nondynamic modeling methods, and nonprimary modeling reports (eg, editorials and reviews). Reviewers extracted the data using a standardized form and assessed the quality of reporting using the STRESS (Strengthening the Reporting of Empirical Simulation Studies) guidelines. A narrative synthesis was conducted for the included studies.

**Results:**

The search identified 1574 studies, with 22 studies meeting the inclusion criteria, including 15 System Dynamics models, 6 agent-based models, and 1 microsimulation model. The studies primarily targeted populations in Australia and the United States, with some focusing on hypothetical scenarios. The models addressed various interventions ranging from specific clinical and health service interventions, such as mental health service capacity increases, to broader social determinants, including employment programs and reduction in access to means of suicide. The studies demonstrated the utility of dynamic models in identifying the synergistic effects of combined interventions and understanding the temporal dynamics of intervention impacts.

**Conclusions:**

Dynamic modeling of suicide and suicide-related behaviors, though still an emerging area, is expanding rapidly, adapting to a range of questions, settings, and contexts. While the quality of reporting was overall adequate, some studies lacked detailed reporting on model transparency and reproducibility. This review highlights the potential of dynamic modeling as a tool to support decision-making and to further our understanding of the complex dynamics of suicide and its related behaviors.

**Trial Registration:**

PROSPERO CRD42022346617; https://www.crd.york.ac.uk/prospero/display_record.php?RecordID=346617

## Introduction

Suicide is a global public health priority; both the Global Mental Health Action Plan and the Sustainable Development Goals include targets to decrease suicide mortality by one-third by 2030 [1]. While there has been a consistent and significant decrease in suicide mortality rates between 1996 and 2016 [2], more than 700,000 people globally died by suicide in 2019. Suicide remains among the top ten leading causes of death globally for young adults aged 15 to 29 years [3]. Nevertheless, suicide is preventable, and the World Health Organization has called on countries to develop comprehensive suicide prevention policies and programs that are evidence-informed [4].

Suicide and its related behaviors are dynamic, complex, and multifaceted health and social phenomena [5-7]. Suicide is a process that develops through stages—thoughts of suicide, suicide attempt, and death or survival of attempt, sometimes followed by reattempt [8]—that are not necessarily unidirectional. Suicide and its related behaviors are influenced by multiple levels of factors within the individual such as biology and genetics, as well as social relationships, and by the family, community, and structural ecosystems [9]. Suicide-related behaviors develop over time and are affected by the interactions between different domains and levels of influence. Path dependence, where past experiences, like adverse childhood events [10], also impact future suicide risk, further underscores the importance of a life course perspective. The dominant paradigm in suicide research has focused on a reductionist, risk factor–driven approach, attempting to identify salient, independent predictors of suicide-related behaviors [11]. While useful for understanding isolated components of a complex system, this approach often misses important insights arising from the larger system of interacting factors.

Systems science, which includes methodologies well-suited for phenomena involving nonlinearity, feedback loops, networks, path dependence, and threshold effects [12], seeks to understand these dynamics [13,14]. Systems science may be particularly useful for identifying how combinations of interventions and policies interact to affect outcomes of interest [15].

Dynamic simulation modeling is a powerful systems science approach for addressing complex public health issues like suicide. These mathematical or computational models are designed to simulate the behavior of complex systems over time. They are especially useful for modeling interactions among system components, improving understanding of the system’s vulnerabilities and strengths, and testing “what-if” scenarios [14]. Dynamic models include various analytical approaches, each with unique strengths for addressing aspects of suicide-related behaviors. System Dynamics (SD) models focus on feedback loops and system-level behavior, making them well-suited for exploring population-level policy impacts. Agent-based models (ABMs) simulate the actions of individual agents and their interactions, allowing for a more granular analysis of heterogeneous populations and network effects. Discrete event simulation is particularly useful for resource-limited systems with structured workflows and where changes occur at discrete points in time. Microsimulation models track individuals over time and interactions. Dynamic simulation models have been widely applied across fields such as business, economics, and health care, and are valuable for addressing complex public health issues [12-14].

Although dynamic simulation models have been used in public health to model disease outbreaks and guide public policy [16], their application to complex mental health outcomes, like suicide and self-harm, remains nascent [17]. Previous reviews have found evidence of dynamic modeling applications to mental health and substance use disorders [17-24], particularly in depression [17-19] and opioid use [21,22]. However, only one scoping review has focused specifically on suicide [25]. This review identified ten studies on suicide interventions and highlighted challenges related to implementation and model confidence, but the review was not systematic and missed many relevant studies. An updated systematic search and comprehensive synthesis of the literature can provide further insights into the application, utility, and value of dynamic modeling for informing suicide and its prevention. Therefore, the objective of this study was to systematically review existing dynamic models of population-level suicide and suicide-related behaviors and summarize their modeling approaches, uses, and outcomes.

## Methods

### Search Strategy

We searched the following eight databases from inception to July 2023: MEDLINE, Embase, PsycINFO, Scopus, Compendex, ACM Digital Library, IEEE Xplore Digital Library, and medRxiv. We developed a search strategy in consultation with a research librarian (Katherine Merucci). The search strategies for each database are available in Tables S1-S8 in Multimedia Appendix 1 and include search terms related to suicide and dynamic modeling approaches (eg, “system dynamics,” “agent-based,” and “microsimulation”) or concepts (eg, “mathematical model” and “computer simulation”). The search was limited to English and French studies for our team to be able to review. The reference lists of included studies were also hand-searched. We imported the citations to Covidence software (Veritas Health Innovation) and removed duplicates. Two independent reviewers (GG and BY) conducted the title and abstract screening and the full-text screening. Disagreements were resolved by consensus. The review protocol is registered through PROSPERO (CRD42022346617) and follows the guidelines provided by the PRISMA (Preferred Reporting Items for Systematic reviews and Meta-Analyses) checklist and PRISMA for Abstract checklist, and the PRISMA-S extension for reporting literature searches in systematic reviews [26] (Multimedia Appendices 2-4).

### Study Selection

The inclusion and exclusion criteria for this review are presented in Textbox 1. We included studies that modeled suicide or suicide-related behaviors as a dynamic process among individuals or in the population, using dynamic modeling methods. Studies were excluded if they did not meet these criteria or if they focused on topics outside the scope of this review.

**Inclusion and exclusion criteria for study selection.**

**Inclusion criteria**

- Study topic: suicide or suicide-related behaviors as a dynamic process among individuals or in the population
- Modeling approach: dynamic modeling methods such as compartmental, System Dynamics, agent-based models, discrete-event simulation, or microsimulation modeling techniques
- Study type: primary modeling studies
- Publication language: English or French

**Exclusion criteria**

- Study topic: intraindividual changes in suicide-related behaviors; self-harm with no suicidal intent; physician-assisted suicide, euthanasia, or medical assistance in dying; terrorism (eg, suicide bomb); evolutionary suicide; studies in the domain of microbiology, bioinformatics, and pharmacology (eg, cell suicide)
- Modeling approach: studies that used other modeling methods such as regression models, conceptual models, and static stochastic simulation models (eg, Monte Carlo simulations); individual-level suicide risk prediction models
- Study type: reviews, editorials, commentaries, web reports, and books
- Publication language: other languages

### Data Extraction and Quality of Reporting Assessment

Two reviewers (GG and BY) independently extracted study data using standardized and prepiloted forms. Any discrepancies were resolved by discussion. Information was extracted on study purpose, target population, population subgroups, location, outcome measures of suicide or suicide-related behaviors, intervention or policy, type of dynamic model, model parameters, data sources, software used, results, limitations, and implications. We contacted authors when information was unclear or missing. Following data extraction, the two reviewers (GG and BY) independently assessed the extent to which studies conformed to the recommendations of the STRESS (Strengthening the Reporting of Empirical Simulation Studies) guidelines, a standardized checklist for assessing the reporting and replicability of empirical simulation models [27]. The guideline recommendations include clearly stating the study objectives, providing details on the base run of the model and simulation experiments, providing information on data sources and input parameters, experimentation details, software and hardware-specific implementation information, and code access [27]. Specific versions of the STRESS guidelines are available for System Dynamics (STRESS-SD), discrete-event simulation (STRESS-DES), and ABM simulation (STRESS-ABS) modeling. The latter was also used for microsimulation modeling, in the absence of a guideline for this method. Disagreements were resolved by consensus. Results were compiled in a table by type of dynamic modeling technique (ie, SD, ABMs, discrete-event simulation, microsimulation modeling) and were summarized narratively.

## Results

### Overview

**Figure 1:**
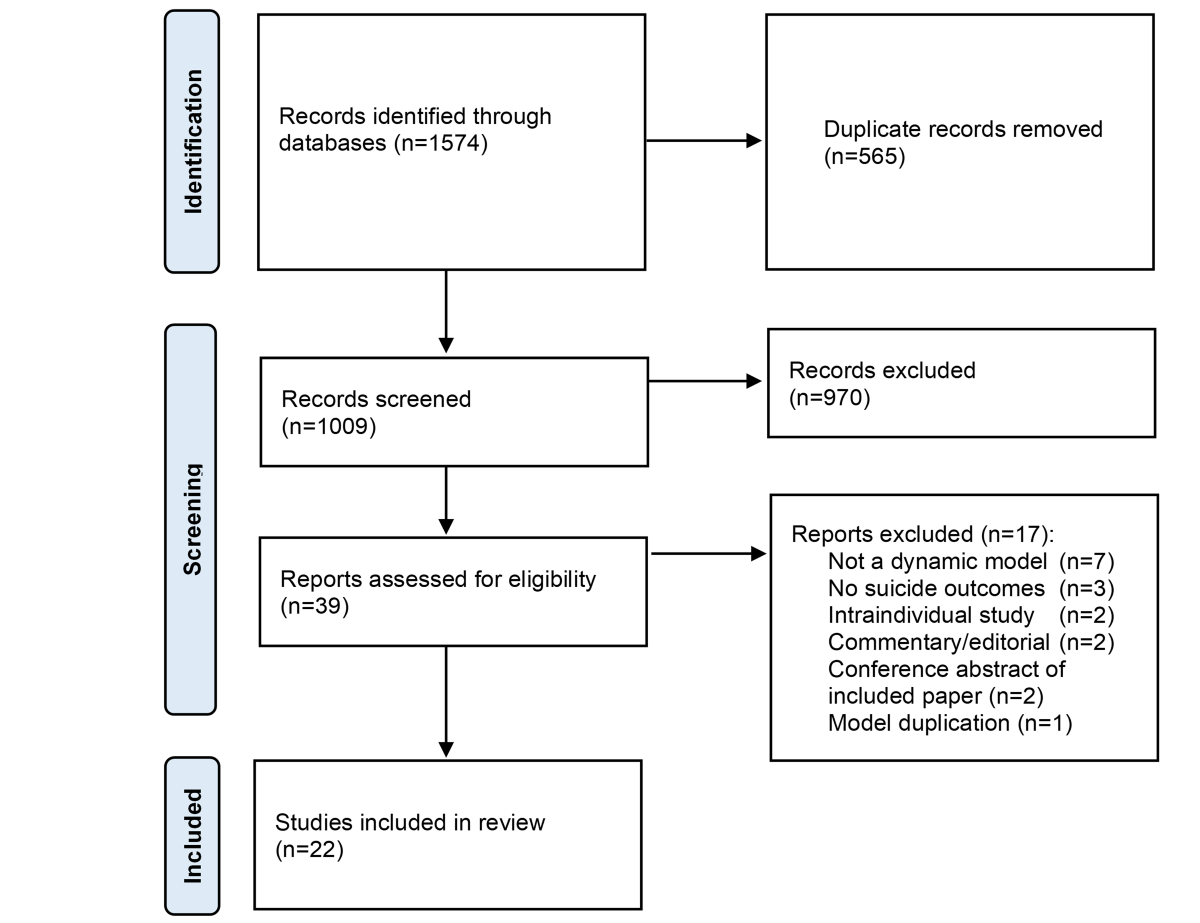
Flowchart of the study selection.

The study selection flowchart is presented in Figure 1. The database search identified 1574 studies from which we excluded 1535 studies following deduplication (n=565) and title and abstract screening (n=970). The number of studies identified from individual databases is available in Tables S1-S8 in Multimedia Appendix 1. Of the remaining 39 studies, we excluded a further 17 studies because they did not use a dynamic model approach (n=7), did not model suicide as an outcome (n=3), were commentary or editorial pieces (n=2), modeled intraindividual changes in suicide behaviors (n=2), were conference abstracts of included papers (n=2), or used the same model and outputs [28] as another included study [29] (n=1). Our final selection included 22 studies. The studies were published between 2009 and 2023, with 80% published in the last 5 years.

### Study Characteristics

The characteristics of the included studies by modeling approach are summarized in Table S9 in Multimedia Appendix 1. About two-thirds of studies (n=15, 68%) used SD or compartmental modeling [29-43], the other third (n=6, 27%) used ABMs [44-49], and one study used microsimulation [50]. No discrete-event simulation models were identified and none of the studies used a hybrid modeling technique. About a third of studies (n=8, 36%) [30,31,34-37,40,41] reported using a participatory approach to model building, of which all were SD studies.

### Geographic Context and Target Population

Most studies modeled populations from specific geographic regions (n=18, 82%), except for 4 studies where authors considered hypothetical simulated settings without assuming any geographic context [44,47-49] (Table S9 in Multimedia Appendix 1). Over half of the studies (n=12, 55%) targeted general populations in Australia [30,31,34-43], including at the national level [38,39] and regional and subregional populations of New South Wales [30,31,34-36,40,41,43], Victoria [42], and Western Australia [37]. Four studies considered populations in the United States, including at the national level [43,50], and locally in New York City [45,46] and Perry County [29]. The remaining models focused on populations in Greece [33], Spain [32], Ukraine [29], and Iran [29]. Six studies modeled suicide at the country-national level for Australia [38,39], Greece [33], Spain [32], and the United States [50].

### Model Structure and Suicide-Related Components

All studies (n=22, 100%) included suicide deaths as a model output, and half further (n=12, 54%) included suicide attempts, defined as suicide attempt hospitalizations [30,38,40], self-harm hospitalizations [34,36,39,41-43], or suicide attempts [31,35,50]. In addition, suicide-related ideation was modeled in six studies [38,39,45-47,50], suicide planning in four studies [38,39,45,46], and suicide risk was modeled in the form of zero risk, prerisk (having risk factors), at-risk (having ideated or planned a suicide), and high-risk (having attempted suicide at least once in their lives) in 2 studies [32,33]. Further, the lethality of suicide attempts was considered in 8 studies [31,34,37-39,41-43] and the method or means of suicide was considered in 5 studies [38,39,45-47], including poisoning [38,39,47], hanging [38,39], firearms [38,39,45,46], drowning [38,39], gases or vapor [38,39], jumping [38,39,47], cutting [38,39], and combined methods [38,39].

Beyond suicide behaviors and deaths, a majority of studies (n=17, 77%) considered poor mental health as part of their model structure, incorporating markers of psychological distress [30,31,34-37,40-43] or mental disorders and substance use or related harms [21,29,32,33,44,46,50]. Moreover, 68% (n=15) of studies constructed their model to capture wider social and structural determinants of suicide-related behaviors, including mental health services [30,31,34,35,37,40-43,45,46], mental health recovery [30,40], early life circumstances [34,36], intimate partner violence [34,36], social support [49], homelessness [34,36], unemployment [34,36,42,43], education [42,43,45,46], income [45,46,50], marital status [45,46], race and ethnicity [45,46,49,50], firearm ownership [45,46], arrest and sentencing [45,46], incarceration [29,45,46], and the COVID-19 pandemic [37,42,43].

### Data Sources

Table 1 presents the parameter data sources across studies. Data to model the target population came mainly from survey and census data (eg, Australian Bureau of Statistics, US Census, and Spanish National Statistics Institute). Data to inform intervention effects mostly came from previously published literature, including systematic reviews, randomized controlled trials, and observational studies, or were modeled using assumptions or a range of effects. Specifically, 91% (n=20) of studies relied on previously published literature as input data sources [29-46,49,50], 86% (n=19) of studies on empirical data from surveys or census [29-43,45,46,49,50], and 55% (n=12) [29-31,34-42] of studies on expert opinions for model parameterizations. About 45% (n=10) of studies derived their parameter values through calibration [35,36], direct calculations [32,33,45,46], or assumptions [44,47,48] by the authors.

**Table 1:** Summary of parameter data sources in dynamic models of suicide-related behaviors.

Parameter data source	Studies (n=22), n (%)	References
Empirical data	19 (86)	[29-43,45,46,49,50]
Literature	20 (91)	[29-46,49,50]
Expert opinion	12 (55)	[29-31,34-42]
Calculated by authors	4 (18)	[32,33,45,46]
Calibrated by authors	2 (9)	[35,36]
Assumed by authors	3 (14)	[44,47,48]

### Tested Interventions and Scenarios

Studies used dynamic models to investigate a range of 43 different suicide prevention interventions and strategies (Table 2). Interventions were predominantly aimed at mental health care, including suicide-specific prevention interventions (n=8, 40%) [31,35-38,40-42], mental health service interventions (n=11, 50%) [30,31,34-38,40-43], increases in mental health service capacity (n=9, 41%) [30,31,34,35,37,40-43], and specific clinical and pharmacological interventions (n=5, 23%) [29,38,39,44,50], as well as strategies to improve social determinants (n=6, 30%) [35-37,40-42] and reduction in access to means of suicide and lethality (n=3, 15%) [38,45,46]. Half of the studies (n=11, 50%) further explored optimal combinations of interventions for suicide prevention [30,31,35-38,40-43]. In addition, dynamic models were used to study the role of social influence (n=4, 20%), including interpersonal loss, depression contagion, and copycat suicide dynamics [44,47-49] on suicide behaviors and to uncover potential underestimation of at-risk populations and suicide deaths at the population-level (n=2, 10%) [32,33].

**Table 2:** List of modeled interventions in suicidal behavior studies using dynamic models.

Intervention			Studies (n=22), n (%)		References
**Suicide-specific prevention interventions (n=8, 36%)**					
	General practitioner training	6 (27)		[31,35,37,38,40,41]	
	Postsuicide attempt care	8 (36)		[31,35-38,40-42]	
	Postdischarge peer support	1 (5)		[36]	
	Suicide helpline services	2 (9)		[31,40]	
	Community-based education programs	2 (9)		[35,37]	
	Awareness campaigns	2 (9)		[41,42]	
	Safety planning	3 (14)		[35,37,41]	
**Mental health service interventions (n=11, 50%)**					
	Mental health hospital to home service	1 (5)		[40]	
	Standard telehealth	1 (5)		[34]	
	Technology-enabled care coordination	4 (18)		[34,36,37,40-42]	
	Web-based mental health services	2 (9)		[31,40]	
	Community-based acute mental health care services	5 (23)		[31,35-37,41]	
	Community-based subacute mental health care services	1 (5)		[40]	
	Re-engagement of individuals lost to services	3 (14)		[30,31,40]	
	Youth early intervention mental health services	1 (5)		[40]	
	Safe space services	3 (14)		[35,37,41]	
	Mental health education programs	1 (5)		[31]	
	Family education and support	3 (14)		[35,37,41]	
	School mental health literacy programs	1 (5)		[38]	
	Direct access to mental health care professionals	1 (5)		[43]	
**Increases in mental health service capacity (n=8, 36%)**					
	Hospital staffing increase	3 (14)		[30,31,40]	
	Mental health service increase	5 (23)		[30,31,34,40,42]	
	GP^a^ mental health service increase	4 (18)		[35,37,41,42]	
	Mental health assessment capacity increase	2 (9)		[30,40]	
	Psychiatrists and allied health services increase	5 (23)		[35,37,41-43]	
	Psychiatric hospital care capacity increase	3 (14)		[35,37,41]	
	Community mental health care services increase	4 (18)		[35,37,41,42]	
	Psychiatric bed decrease	1 (5)		[30]	
**Specific clinical and pharmacological interventions (n=5, 23%)**					
	Opioid agonist treatment interventions	1 (5)		[29]	
	Transcranial magnetic stimulation	1 (5)		[44]	
	Brief-contact intervention in hospital	1 (5)		[38]	
	Psychosocial treatment approaches	2 (9)		[38,39]	
	Antidepressant treatment	1 (5)		[50]	
**Improvements in social determinants (n=6, 27%)**					
	Reducing childhood adversity	1 (5)		[36]	
	Addressing youth unemployment	2 (9)		[36,42]	
	Reducing unemployment	2 (9)		[36,42]	
	Reducing domestic violence	1 (5)		[36]	
	Reducing homelessness	1 (5)		[36]	
	Community support programs	1 (5)		[40]	
	Community infrastructure spend per annum	1 (5)		[40]	
	Social connectedness programs	4 (18)		[35-37,41]	
**Reduction in access to means of suicide and lethality (n=3, 14%)**					
	Firearms restrictions	2 (9)		[45,46]	
	Reduction in method lethality	1 (5)		[38]	

^a^GP: general practitioner.

### Timeline and Time Unit

The time horizon for the model outputs varied between 3.5 and 30 years, ranging between the years 2000 and 2041 (Table S9 in Multimedia Appendix 1). The time unit was reported in 59% (n=13) of studies. Among these, seven studies used a daily [44] or subdaily model [31,34-37,41], one study used a weekly model [50], one study used a monthly model [49], two studies used a half-yearly model [32,33], and two studies used a yearly model [45,46].

### Software Implementation

The computational platform or software used to conduct the studies is given in Table S9 in Multimedia Appendix 1. Most studies (n=15, 68%) provided their computational platform. Among those that applied SD modeling, all nine studies used Stella Architect [30,31,34-37,40,42,43]. Among those that used agent-based modeling, two studies used REPAST for Java and Eclipse [44,45], one study used AnyLogic [46], one study used Netlogo [48] and one study used Borland C++ (Borland Software Corporation) [49]. The study that applied microsimulation modeling used REPAST Simphony [50].

### Calibration and Validation

Over half of the studies (n=12, 54%) reported calibrating their model against empirical data [30,31,34,35,38,40-43,45,46,50]. Of these, six studies reported specific calibration methods including Powell’s method [31,34-36,41,43] and a Bayesian framework [29]. Model validation was conducted in 64% (n=14) of studies [30,31,34-43,45,46] and included external validity by comparing model outputs to real-world historical data or face validity through expert and stakeholder consultations. Moreover, most studies (n=15, 68%) conducted sensitivity analyses on interventions or key parameters with uncertain baseline assumptions to check the robustness of their model to value changes [30-37,40-42,45,46,48,50].

### Study Results

The studies evaluated a wide range of potential interventions (Table 2 and Table S10 in Multimedia Appendix 1), generally reporting a favourable impact on suicide and suicidal behavior prevention, alone or in combination, including postsuicide attempt care (n=8) [31,35-38,40-42], technology-enabled care coordination (n=5) [34,35,37,41,42], improving social connectedness (n=4) [35,37,40,41], increasing mental health service capacity (n=4) [30,42,43,50], service re-engagement (n=3) [30,31,40], community support (n=2) [31,37], family education and support (n=2) [37,41], employment programs (n=2) [35,42], firearm disqualifications (n=2) based on a history of drug or alcohol misdemeanors [45] or based on psychiatric hospitalization or mental health treatment [46], safety planning (n=1) [41], general practitioner (GP) training (n=1) [38], reducing childhood adversity (n=1) [35], reducing psychological distress (n=1) [40], psychosocial therapy (n=1) [39], opioid agonist treatment scale up (n=1) [29], transcranial magnetic stimulation for depression (n=1) [44], and antidepressant treatment (n=1) [50]. The main findings of each included study are summarized in Table S10 in Multimedia Appendix 1.

### Study Limitations

Authors acknowledged a number of limitations with their use of dynamic modeling (Table S10 in Multimedia Appendix 1), most frequently citing potential measurement error in the data sources used to parameterize and calibrate their models [30,31,34-38,40,42,45,46,48]; under-enumeration of the number of suicide attempts and cases in the population [30,31,34-38,40,42]; lack of generalizability of findings to other contexts [31,34-38,41,45,46]; missing individual mechanisms, trajectories, or social influences [30,38,40,46,48], analysis of a limited set of interventions [30,38,40,41], and uncertainties around model assumptions [39,45,46,48]. Some authors further noted that some simulated scenarios were not specifically tied to any direct intervention or program. For instance, Occhipinti et al [36] found that in their model, a 20% to 50% reduction in childhood adversity had the most significant impact on suicide prevention but acknowledged that no program targeting childhood adversity was specifically modeled. Some authors also emphasized the importance of exercising caution when interpreting results. For example, in their ABM of New York City, Cerdá et al [45] found that in their model, denying firearm access based on a history of drug and alcohol misdemeanors had the greatest impact on preventing firearm suicide among people with a prior history of alcohol misdemeanors, but cautioned that these findings needed to be balanced with the potential for creating additional forms of marginalization for these already vulnerable populations.

### Quality of Reporting

Table 3 presents an overview of the quality of reporting in the suicide dynamic modeling literature using the STRESS guidelines. Detailed assessments are also provided in Tables S11, S12, and S13 in Multimedia Appendix 1 for studies using SD modeling, ABMs, and microsimulation, respectively. All studies explained the purpose and background of their model and provided details about their base model logic [29-50], and most presented a base model overview diagram to help describe their model to readers (n=17, 77%) [31-46,50]. Among the 20 studies that included an experimentation aim to their model [29-31,34-50], nearly all (n=19) provided a clear description of the tested interventions, policies, or scenarios and a rationale for their selection [29-31,34-43,45-50], and all included details on the scenario logic by explaining the difference between the base case and the tested scenario. The majority of studies provided details about their model inputs, including a description of the input parameters and their values (n=19, 86%) [29,31-37,40-50]; their model outputs, including details about their outcome variables and how they were calculated (n=19, 86%) [29-38,40-43,45,46,48-50]; their data sources (n=18, 82%) [29-37,40-46,49,50]; their estimation approach (n=16 of 20 studies where applicable, 80%) [29-31,34-37,40-43,45,46,48-50]; and their algorithms (n=15, 68%) [29,31-37,41,43,45-47,49,50]. While the model execution was well reported in the ABM studies (n=5 out of 6 ABM studies) [44-46,48,49] and the microsimulation study [50], none of the SD studies reported their integration method, such as the Euler or Runge-Kutta methods [51]. Finally, only one study made their code available [48] while none provided system specifications, such as model run time and hardware, that may be useful to evaluate computational power needs.

**Table 3:** Percentage of suicidal behavior studies (n=22) using dynamic models that meet common recommendations to dynamic models based on STRESS^a,b^.

Recommendation	Yes, n (%)	No, n (%)	N/A^c^, n (%)
Purpose of the model	22 (100)	0 (0)	0 (0)
Base model logic	22 (100)	0 (0)	0 (0)
Assumptions	20 (91)	2 (9)	0 (0)
Scenario logic	20 (91)	0 (0)	2 (9)
Model output details	19 (86)	3 (14)	0 (0)
Input parameter details	19 (86)	3 (14)	0 (0)
Experimentation aims	19 (86)	1 (5)	2 (9)
Data sources	18 (82)	4 (18)	0 (0)
Base model overview	17 (77)	5 (23)	0 (0)
Estimation approach	16 (73)	4 (18)	2 (9)
Software used	16 (73)	6 (27)	0 (0)
Algorithms	15 (68)	7 (32)	0 (0)
Run length and time step	14 (64)	8 (36)	0 (0)
Initialization	14 (64)	7 (32)	1 (5)
Model execution	6 (27)	16 (73)	0 (0)
Modeling code availability	1 (5)	21 (95)	0 (0)
System specification	0 (0)	22 (100)	0 (0)
Random sampling algorithm	0 (0)	3 (14)	19 (86)
Preprocessing details	0 (0)	0 (0)	22 (100)

^a^STRESS: Strengthening the Reporting of Empirical Simulation Studies.

^b^Full details on the quality of reporting of individual studies are available in Tables S11-S13 in Multimedia Appendix 1.

^c^Not applicable.

## Discussion

### Principal Findings

This systematic review provides an in-depth synthesis of the application of dynamic simulation models in suicide research. We identified 22 studies that applied SD (n=15), ABMs (n=6), and microsimulation (n=1) to investigate the impact of 43 different suicide-related strategies and scenarios, alone or combined, ranging from suicide-specific clinical and health service interventions to addressing broader social factors and access to means of suicide, as well as the role of social influence and clustering. While the application of dynamic modeling to suicide and suicide-related behaviors is still limited, this review points to a growing field with an increasing diversity of models adapted to different questions, settings, contexts, and perspectives. Our review demonstrates that dynamic modeling offers an important tool not only to facilitate direct decision-support analysis and navigate complex decision-making but also to assist in understanding the dynamic process in which suicide-related behaviors are embedded. This is consistent with other complex public health phenomena where systems science is being successfully used, such as the opioid crisis [21,22], injury and violence prevention [24], and smoking behaviors [23].

### The Value of Dynamic Models in Suicide Research

#### A Nuanced Understanding of Interventions

The body of work found in this review illustrates the wide applicability of dynamic modeling to a range of interventions and scenarios. Unlike traditional approaches that focus on one intervention or factor at a time, the ability of dynamic modeling to consider the interacting and nonlinear effects of interventions on a system as a whole and through time can be a powerful tool to uncover limits and trade-offs under different scenarios and inform both short and long-term policy and program goals. The studies show that suicide prevention strategies can have synergistic and cumulative effects that can be optimally combined to optimize resources [30,31,35-38,40-42], and their intensity and scale [30,36,39,40], timing [39,42], and duration [36,37] can be explored to gain insight into what interventions may have the greatest impact on suicide prevention. For instance, Occhipinti et al [37] examined 286 possible scenarios based on the combination of 13 interventions and modeled for both a short-term (5 years) and long-term (20 years) horizon. They found that the best-performing combinations differed between the short and long-term horizons, showing that some interventions, such as community support programs aimed at enhancing social connectedness, may be slower in their impact but have amplifying effects over time, while others may have rapid but plateauing impacts. Occhipinti et al [36] similarly reported delayed but amplifying effects for interventions that target improvements in social determinants compared with quick but plateauing effects for specific mental health and suicide prevention initiatives. Dynamic models can also provide insight into implementation timing. For example, Vacher et al [42] found that postsuicide attempt care was the most impactful intervention for suicide prevention*,* but that delay in its implementation reduced the strength of its impact over time.

In addition, dynamic modeling can provide a better understanding of both the intended and unintended consequences of suicide prevention actions. Some of the studies from collaborators in Australia showed that well-intentioned interventions may counterintuitively provide little benefit and even negative effects. For instance, Atkinson et al [31] found that GP training combined with mental health education programs unexpectedly increased self-harm hospitalizations and suicide deaths. Page et al [40] also found that GP training had minimal impact on suicide attempts and deaths unless combined with an increase in mental health care capacity. Similarly, Vacher et al [43] found that direct patient access to mental health services would increase self-harm hospitalizations and suicide in Australia if it was not concurrently combined with a greater growth rate of mental health service capacity. Finally, Vacher et al [42] reported that public health awareness programs implemented alone would lead to more self-harm hospitalizations and suicide. Results from their model suggested that such campaigns would tax the mental health service system, thereby reducing access and leading individuals to feel discouraged and at increased risk for psychological distress and suicide. These findings underscore the complexity of suicide prevention efforts and emphasize the key role that dynamic modeling can play in the development of more nuanced strategies that can address the unique challenges in suicide prevention.

#### A Nuanced Understanding of Contexts, Populations, and Perspectives

Evidence from the 22 studies in this review also demonstrates the adaptability of dynamic models to understand how scenarios and strategies might perform in different contexts [29,32-34,36,47] and populations [45,46]. For example, Degenhardt et al [29] found that opioid agonist treatment scale-up strategies may reduce suicide deaths in people who use drugs across three different settings in the United States, Ukraine, and Iran, but that the impact would vary depending on the major cause of deaths in each region. Modeling different public health contexts, Iorfino et al [34] found that technology-enabled care coordination would be equally likely to reduce suicide-related behaviors in a typical public mental health context as in a crisis context resulting from the COVID-19 pandemic in Australia. In addition to adapting existing models from one population to another [30,33,34,37,39,41,45], some studies also developed their model to explore outcomes for specific subgroups [31,35,38,39,42-46,50]. For example, Keyes et al [46] found that firearm disqualifications based on a history of psychiatric treatment may have little impact on the population-wide suicide rates of New York City, but a significant impact among the high-risk group with a history of mental health treatments.

The impact of different perspectives [29,32-34,36,47] and decision-making priorities on suicide outcomes [36,41] were also explored using dynamic modeling. For example, Skinner et al [41] compared the regional planning perspective with the state-level perspective across ten regions of New South Wales in Australia, modeling different combination scenarios from 13 possible interventions. They found that the regional-specific optimal combinations would lead to a greater overall reduction in suicide deaths than the optimal combinations at the state level. Occhipinti et al [36] similarly reported that competing priorities between the federal and state levels presented a marked trade-off between minimizing suicide deaths and minimizing service disengagement in New South Wales. These findings show the potential of dynamic modeling to provide insights on population-level suicide both for specific contexts and across contexts, perspectives that are often needed for optimal and sustainable strategy planning and coordination.

#### An Expandable Tool

Finally, dynamic models of suicide and their underlying systems science approach differ from most conventional statistical approaches in that they are expandable and can be built upon as needed. They can be revised and expanded to capture greater details of the system, updated to represent novel and emerging interventions and scenarios like the COVID-19 pandemic, and modified with structures and parameters that reflect specific populations, contexts, priorities, and timelines. A third of the studies (n=7) in this review [30,33,34,37,39,41,45] report building their model from previously published models, illustrating the plasticity of dynamic models to integrate system complexities and specificities. One illustrative example is the body of work from a group of researchers in Australia who extended and adapted their previous models to new research questions. For instance, the researchers used an SD model built for one region in the state of New South Wales (Hunter New England and Central Coast) [36] and adapted it to ten regions in that state [41] and to a specific population of Perth South, Western Australia [37]. The researchers also incorporated greater system complexity in their models over time across their publications, starting from a single-component dynamic structure of suicide-related behaviors [38] to a large multi-component structure [43] that includes (1) population, (2) education, (3) employment, (4) developmental or psychopathological vulnerability, (5) psychological distress, (6) mental health services, (7) suicidal behavior, (8) and the impacts of the COVID-19 pandemic.

### Quality of Evidence

While the quality of reporting was overall adequate, many of the studies did not provide details of their input parameters, data sources, estimation approach, model validation, sensitivity analysis, or supplementary materials, and none provided their code or a computer model sharing statement. Model transparency and reproducibility are critical components of advancing the use of simulation models in systems science, and as such, documenting and reporting the development of models should be considered essential [27,52]. We also found that only a minority of studies (n=8, 40%) engaged in a participatory approach for model building in partnerships with stakeholders and decision makers [30,31,34-37,40,41], which could limit the potential usefulness and feasibility of the models for decision-making.

### Strengths and Limitations

This review is the first to provide a systematic overview of dynamic modeling in suicide research, its applications, and the various contexts in which it has been used. We applied a rigorous systematic approach to the literature search, identifying several studies not included in previous reviews. By summarizing the different modeling approaches in the literature, the review can illuminate promising avenues for future research and highlight areas where dynamic simulation models can be particularly useful.

Several limitations of this systematic review are also worth noting. In addition to restricting the search criteria to English and French languages, we did not search the gray literature for nonpeer-reviewed papers and reports. However, our search identified studies in line with those from a previous scoping review on simulation models for suicide prevention [25] and added several previously unidentified studies to this work. We evaluated the quality of studies using a quality reporting tool but did not directly evaluate the risk of bias in the studies. Nonetheless, this approach has been successfully used in previous systematic reviews of health simulation models [16,21]. Our review focused on dynamic models of suicide applied at the population level, but we recognize that dynamic models could also be useful for studying intraindividual dynamics of suicide-related behaviors that could be informative to public health and policies. Finally, the heterogeneity in modeling approaches, coupled with disparities in data quality and availability across studies, may limit the ability to draw generalized conclusions for future work.

### Future Research

#### Overview

The studies included in this review proposed a number of research directions based on their work (Table S10 in Multimedia Appendix 1), including adapting their model to other populations, regions, and contexts [32-35,38,39,41,45,46], testing other scenarios and interventions [35,40,41], and enhancing the model with additional components or dimensions [46,47,49]. Building on these and the current literature, we identified several avenues for future work in the dynamic modeling of suicide.

#### Modeling Implementable Interventions and Policies

First, future work should aim to model suicide prevention strategies that reflect actionable interventions and policies. This aligns with the recent commentary by Caine [53], who applauds the promise of dynamic modeling for suicide prevention, but calls for the modeling of measurable implementation strategies, such as available social support measures, and for better integrating the current organization and coordination of primary, emergency, and follow-up health care systems into models. Participatory model-building with stakeholders and decision makers may further help to identify practical leverage points and interventions relevant to their specific context [54].

#### Stratification by Subgroups and Key Variables

Second, future models should consider modeling results by age, sex, gender, means of suicide, and other equity-relevant variables, as determinants, interventions, and outcomes vary among these groups. The World Health Organization stresses the need to collect and report on suicide surveillance data disaggregated by age, sex, and means of suicide [4]. However, only a few studies in this review presented their results by sex [38,39,44,50] or age groups [35,42,43]. Even fewer considered characteristics such as Indigenous identity [31], rural residence [42], or membership in a high-risk group, such as those who have received mental health treatment in the past year [46] or those with prior alcohol- or drug-related arrests [45]. Additionally, stratification by environmental and geographic factors, such as temperature, air pollution, and local topography, which have shown associations with suicide risk [55-58], could provide more nuanced insights across different contexts. Though incorporating such data can be complex and may require the integration of stochasticity, it could provide more realistic and actionable insights, given the highly patterned nature of suicide by these characteristics. This approach would also allow the evaluation of the potential emergence and intensification of disparities among populations, and help avoid unintended consequences.

#### Integrating Broader Social and Structural Determinants and Interventions

Third, future studies should aim to integrate broader social and structural determinants of suicide-related behaviors as part of their model. Health care service interventions and capacity growth remain important levers for suicide prevention and were the focus of most studies in this review (Table 2). However, a broader socioecological lens to suicide prevention is also needed to address both downstream and upstream risk factors and promote whole-of-population mental health [59,60]. As shown in studies from this review, the effects of addressing social determinants, such as social support and unemployment, may be harder to identify in the short term but have cumulative and broad effects in the long term [36,37,42]. A life course perspective is also vital due to the significant impact of adverse experiences, particularly in childhood [36]. Within a framework of short-term decision-making processes, the ability of dynamic models to simulate long-term effects can be an important tool to identify interventions that could have a significant impact at the population level, aligning immediate actions with broader, sustained outcomes.

#### Integration of Multiple Data Sources

Fourth, the integration of multiple data sources is needed for improving future suicide simulation models. Namely, current models rely on historical data or research findings that do not capture system changes in behaviors or the health system. By integrating diverse and real-time data, such as social media interactions, crisis helpline calls, or near real-time surveillance data, future models can better integrate changing patterns, and offer a timelier analysis of suicide-related behaviors. The use of data science methods, such as machine learning, can further enhance predictive accuracy and the ability to test the potential effects of interventions as conditions change over time [61].

#### Modeling Cost-Effectiveness

Fifth, applying dynamic simulation models to evaluate the cost-effectiveness of different suicide prevention strategies could accelerate the uptake of evidence-based interventions, as cost-benefit trade-offs are often an important consideration in decision-making. This review did not identify any study that included a cost-effectiveness analysis, in contrast to a recent systematic review of simulation models of opioid use and overdose [21], which found that most studies in the field focused on assessing the cost-effectiveness of various strategies to address the opioid overdose crisis. With dynamic models, researchers and decision makers can explore different scenarios, considering both direct and indirect costs and potential auxiliary benefits over various time frames.

### Conclusions

This review shows dynamic simulation modeling as an emerging and transformative tool in suicide research and decision-making. The current literature demonstrates the broad applicability of dynamic modeling across various interventions and scenarios, adaptable to different contexts, populations, and perspectives. The insights gathered through these models have the potential to help decision makers navigate complex scenarios and invest in strategies that promise the most significant impact for reducing suicide and suicide-related behaviors over various timeframes. Building on this body of knowledge involves refining models to include broader social and structural determinants and integrating multiple sources of data, including cost data, to better inform decisions. As resources continue to be constrained and the need for effective interventions grows, the role of dynamic simulation models will undoubtedly remain pivotal in shaping evidence-based suicide prevention policies and strategies for the future.

## Appendix

Multimedia Appendix 1 Supplementary tables.

Multimedia Appendix 2 PRISMA checklist.

Multimedia Appendix 3 PRISMA abstract checklist.

Multimedia Appendix 4 PRISMA-S extension for systematic review checklist.

## Abbreviations

ABM: agent-based model
GP: general practitioner
PRISMA: Preferred Reporting Items for Systematic reviews and Meta-Analyses
SD: System Dynamics
STRESS: Strengthening the Reporting of Empirical Simulation Studies

## Data Availability

The data sets generated and analyzed during this study are available from the corresponding author upon reasonable request.

## References

[1] (2021). Comprehensive Mental Health Action Plan. World Health Organization.

[2] Naghavi M, Global Burden of Disease Self-Harm Collaborators (2019). Global, regional, and national burden of suicide mortality 1990 to 2016: systematic analysis for the global burden of disease study 2016. BMJ.

[3] (2021). Suicide Worldwide in 2019: Global Health Estimates. World Health Organization.

[4] (2021). Live Life: An Implementation Guide for Suicide Prevention. World Health Organization.

[5] Thompson LH, Lang JJ, Olibris B, Gauthier-Beaupré A, Cook H, Gillies D, Orpana H (2020). Participatory model building for suicide prevention in Canada. Int J Ment Health Syst.

[6] de Beurs D, Bockting C, Kerkhof A, Scheepers F, O'Connor R, Penninx B, van de Leemput I (2021). A network perspective on suicidal behavior: understanding suicidality as a complex system. Suicide Life Threat Behav.

[7] de Beurs D, de Winter RF, Helbich M, Bockting C (2023). Suicidal behavior from a complex system perspective: individual, dynamical, and contextual. Suicide Risk Assessment and Prevention.

[8] Klonsky ED, May AM, Saffer BY (2016). Suicide, suicide attempts, and suicidal ideation. Annu Rev Clin Psychol.

[9] Ullman K, Landsteiner A, Linskens E, MacDonald R, McKenzie L, Murdoch M, Sayer N, Stroebel B, Sultan S, Venables N (2021). Risk and Protective Factors Across Socioecological Levels of Risk for Suicide: An Evidence Map. Department of Veterans Affairs (US).

[10] Dube SR, Anda RF, Felitti VJ, Chapman DP, Williamson DF, Giles WH (2001). Childhood abuse, household dysfunction, and the risk of attempted suicide throughout the life span: findings from the adverse childhood experiences study. JAMA.

[11] Olarte-Godoy J (2022). Newtonian science, complexity science and suicide-critically analysing the philosophical basis for suicide research: a discussion paper. J Adv Nurs.

[12] Luke DA, Stamatakis KA (2012). Systems science methods in public health: dynamics, networks, and agents. Annu Rev Public Health.

[13] Carey G, Malbon E, Carey N, Joyce A, Crammond B, Carey A (2015). Systems science and systems thinking for public health: a systematic review of the field. BMJ Open.

[14] Lich KH, Ginexi EM, Osgood ND, Mabry PL (2013). A call to address complexity in prevention science research. Prev Sci.

[15] Hawton K, Pirkis J (2017). Suicide is a complex problem that requires a range of prevention initiatives and methods of evaluation. Br J Psychiatry.

[16] Jalali MS, DiGennaro C, Guitar A, Lew K, Rahmandad H (2022). Evolution and reproducibility of simulation modeling in epidemiology and health policy over half a century. Epidemiol Rev.

[17] Darabi N, Hosseinichimeh N (2020). System dynamics modeling in health and medicine: a systematic literature review. Syst Dyn Rev.

[18] Davahli MR, Karwowski W, Taiar R (2020). A system dynamics simulation applied to healthcare: a systematic review. Int J Environ Res Public Health.

[19] Long KM, Meadows GN (2018). Simulation modelling in mental health: a systematic review. J Simul.

[20] Naumann RB, Guynn I, Clare HM, Lich KH (2022). Insights from system dynamics applications in addiction research: a scoping review. Drug Alcohol Depend.

[21] Cerdá M, Jalali MS, Hamilton AD, DiGennaro C, Hyder A, Santaella-Tenorio J, Kaur N, Wang C, Keyes KM (2022). A systematic review of simulation models to track and address the opioid crisis. Epidemiol Rev.

[22] Shojaati N, Osgood ND (2021). Dynamic computational models and simulations of the opioid crisis: a comprehensive survey. ACM Trans Comput Healthcare.

[23] Skinner A, Occhipinti JA, Osgood ND (2021). A dynamic modelling analysis of the impact of tobacco control programs on population-level nicotine dependence. Sci Rep.

[24] Naumann RB, Austin AE, Sheble L, Lich KH (2019). System dynamics applications to injury and violence prevention: a systematic review. Curr Epidemiol Rep.

[25] Schuerkamp R, Liang L, Rice KL, Giabbanelli PJ (2023). Simulation models for suicide prevention: a survey of the state-of-the-art. Computers (Basel).

[26] Rethlefsen ML, Kirtley S, Waffenschmidt S, Ayala AP, Moher D, Page MJ, Koffel JB (2021). PRISMA-S: an extension to the PRISMA statement for reporting literature searches in systematic reviews. Syst Rev.

[27] Monks T, Currie CSM, Onggo BS, Robinson S, Kunc M, Taylor SJE (2019). Strengthening the reporting of empirical simulation studies: introducing the STRESS guidelines. J Simul.

[28] Stone J, Degenhardt L, Grebely J, Larney S, Altice FL, Smyrnov P, Rahimi-Movaghar A, Alavi M, Young AM, Havens JR, Miller WC, Hickman M, Vickerman P (2021). Modelling the intervention effect of opioid agonist treatment on multiple mortality outcomes in people who inject drugs: a three-setting analysis. Lancet Psychiatry.

[29] Degenhardt L, Grebely J, Stone J, Hickman M, Vickerman P, Marshall BDL, Bruneau J, Altice FL, Henderson G, Rahimi-Movaghar A, Larney S (2019). Global patterns of opioid use and dependence: harms to populations, interventions, and future action. Lancet.

[30] Atkinson JA, Page A, Heffernan M, McDonnell G, Prodan A, Campos B, Meadows G, Hickie IB (2019). The impact of strengthening mental health services to prevent suicidal behaviour. Aust N Z J Psychiatry.

[31] Atkinson JA, Skinner A, Hackney S, Mason L, Heffernan M, Currier D, King K, Pirkis J (2020). Systems modelling and simulation to inform strategic decision making for suicide prevention in rural new South Wales (Australia). Aust N Z J Psychiatry.

[32] De la Poza E, Jódar L (2018). A short-term population model of the suicide risk: the case of Spain. Cult Med Psychiatry.

[33] De la Poza E, Jódar L, Douklia G (2019). Modeling the spread of suicide in Greece. Complex Syst.

[34] Iorfino F, Occhipinti JA, Skinner A, Davenport T, Rowe S, Prodan A, Sturgess J, Hickie IB (2021). The impact of technology-enabled care coordination in a complex mental health system: a local system dynamics model. J Med Internet Res.

[35] Occhipinti JA, Skinner A, Carter S, Heath J, Lawson K, McGill K, McClure R, Hickie IB (2021). Federal and state cooperation necessary but not sufficient for effective regional mental health systems: insights from systems modelling and simulation. Sci Rep.

[36] Occhipinti JA, Skinner A, Carter S, Heath J, Lawson K, McGill K, McClure R, Hickie IB (2021). Federal and state cooperation necessary but not sufficient for effective regional mental health systems: insights from systems modelling and simulation. Sci Rep.

[37] Occhipinti JA, Rose D, Skinner A, Rock D, Song YJC, Prodan A, Rosenberg S, Freebairn L, Vacher C, Hickie IB (2022). Sound decision making in uncertain times: can systems modelling be useful for informing policy and planning for suicide prevention?. Int J Environ Res Public Health.

[38] Page A, Atkinson JA, Heffernan M, McDonnell G, Hickie I (2017). A decision-support tool to inform Australian strategies for preventing suicide and suicidal behaviour. Public Health Res Pract.

[39] Page A, Atkinson JA, Heffernan M, McDonnell G, Prodan A, Osgood N, Hickie I (2018). Static metrics of impact for a dynamic problem: the need for smarter tools to guide suicide prevention planning and investment. Aust N Z J Psychiatry.

[40] Page A, Atkinson JA, Campos W, Heffernan M, Ferdousi S, Power A, McDonnell G, Maranan N, Hickie I (2018). A decision support tool to inform local suicide prevention activity in greater western Sydney (Australia). Aust N Z J Psychiatry.

[41] Skinner A, Occhipinti JA, Song YJC, Hickie IB (2021). Regional suicide prevention planning: a dynamic simulation modelling analysis. BJPsych open.

[42] Vacher C, Ho N, Skinner A, Robinson J, Freebairn L, Lee GY, Iorfino F, Prodan A, Song YJC, Occhipinti JA, Hickie IB (2022). Optimizing strategies for improving mental health in Victoria, Australia during the COVID-19 era: a system dynamics modelling study. Int J Environ Res Public Health.

[43] Vacher C, Skinner A, Occhipinti JA, Rosenberg S, Ho N, Song YJC, Hickie IB (2023). Improving access to mental health care: a system dynamics model of direct access to specialist care and accelerated specialist service capacity growth. Med J Aust.

[44] Andarlia HT, Gunawan I (2021). An agent-based model of contagion effects in affected depression and its recovery process. J Phys Conf Ser.

[45] Cerdá M, Hamilton AD, Tracy M, Branas C, Fink D, Keyes KM (2022). Would restricting firearm purchases due to alcohol- and drug-related misdemeanor offenses reduce firearm homicide and suicide? An agent-based simulation. Inj Epidemiol.

[46] Keyes KM, Hamilton A, Swanson J, Tracy M, Cerdá M (2019). Simulating the suicide prevention effects of firearms restrictions based on psychiatric hospitalization and treatment records: social benefits and unintended adverse consequences. Am J Public Health.

[47] Liu J, Li L, Russell K (2017). What becomes of the broken hearted? An agent-based approach to self-evaluation, interpersonal loss, and suicide ideation.

[48] Mesoudi A (2009). The cultural dynamics of copycat suicide. PLoS One.

[49] Morabito PN, Cook AV, Homan CM, Long ME, Agarwal N, Xu K, Osgood N (2015). Agent-based models of copycat suicide. Social Computing, Behavioral-Cultural Modeling, and Prediction. SBP 2015. Lecture Notes in Computer Science, vol 9021.

[50] Zhang C, Zafari Z, Slejko JF, Castillo WC, Reeves GM, dosReis S (2023). Impact of undertreatment of depression on suicide risk among children and adolescents with major depressive disorder: a microsimulation study. Am J Epidemiol.

[51] Butcher JC (2016). Numerical Methods for Ordinary Differential Equations.

[52] Martinez-Moyano IJ (2012). Documentation for model transparency. Syst Dyn Rev.

[53] Caine ED (2019). Building the foundation for comprehensive suicide prevention—based on intention and planning in a social-ecological context. Epidemiol Psychiatr Sci.

[54] Claassen CA, Pearson JL, Khodyakov D, Satow PM, Gebbia R, Berman AL, Reidenberg DJ, Feldman S, Molock S, Carras MC, Lento RM, Sherrill J, Pringle B, Dalal S, Insel TR (2014). Reducing the burden of suicide in the U.S.: the aspirational research goals of the national action alliance for suicide prevention research prioritization task force. Am J Prev Med.

[55] Heo S, Lee W, Bell ML (2021). Suicide and associations with air pollution and ambient temperature: a systematic review and meta-analysis. Int J Environ Res Public Health.

[56] Aguglia A, Giacomini G, Montagna E, Amerio A, Escelsior A, Capello M, Cutroneo L, Ferretti G, Scafidi D, Costanza A, Serafini G, Amore M (2021). Meteorological variables and suicidal behavior: air pollution and apparent temperature are associated with high-lethality suicide attempts and male gender. Front Psychiatry.

[57] Merli R, Costanza A (2024). Effectiveness of physical barriers to prevent suicide by jumping from high-risk bridges: from an integrative review to a northern Italian province's paradigm. Prev Med Rep.

[58] Brown A, Hellem T, Schreiber J, Buerhaus P, Colbert A (2022). Suicide and altitude: a systematic review of global literature. Public Health Nurs.

[59] Sher L (2019). Is it time to employ Rose's theorem to prevent suicide?. Aust N Z J Psychiatry.

[60] Iskander JK, Crosby AE (2021). Implementing the national suicide prevention strategy: time for action to flatten the curve. Prev Med.

[61] Wulz AR, Law R, Wang J, Wolkin AF (2022). Leveraging data science to enhance suicide prevention research: a literature review. Inj Prev.

