# Supplementary material for: Dynamic Simulation Models of Suicide and Suicide-Related Behaviors: Systematic Review
Source: JMIR Public Health Surveill. 2024 Dec 2;10:e63195. doi: 10.2196/63195 (PMC11650081; doi:10.2196/63195)
Supplement: Multimedia Appendix 1 [file publichealth_v10i1e63195_app1.docx]

**Table S1. Search strategy and results for MEDLINE from inception to July 13, 2023**

| **#** | **SEARCH TERMS** |
| --- | --- |
| 1 | exp suicide |
| 2 | suicid*.ab |
| 3 | suicid*.ti,kw,kf |
| 4 | or/1-3 |
| 5 | exp *Systems Analysis/ or *systems theory/ or *exp Models, Theoretical/ or exp *decision support techniques/ |
| 6 | (system? thinking or system? science or system? approach* or system? theor* or system? analys* or system? dynamic? or dynamic system? or compartmental model* or mathematical model* or mechanistic model* or dynamic model* or aggregate model* or agent-based model* or differential equation* or monte carlo method* or microsimulation model* ordinary differential or complexity analys* or system? medicine or system? oriented approach* or decision support technique?).tw,kw,kf. |
| 7 | or/5-6 |
| 8 | and/4,7 |
| 9 | limit 8 to (English or French) |
| RESULTS: **267 STUDIES** | |

**Table S2. Search strategy and results for Embase from inception to July 13, 2023**

| **#** | **SEARCH TERMS** |
| --- | --- |
| 1 | exp suicide |
| 2 | suicid*.ab |
| 3 | suicid*.ti,kw,kf |
| 4 | or/1-3 |
| 5 | exp *Systems Analysis/ or *systems theory/ or *exp Models, Theoretical/ or exp *decision support techniques/ |
| 6 | (system? thinking or system? science or system? approach* or system? theor* or system? analys* or system? dynamic? or dynamic system? or compartmental model* or mathematical model* or mechanistic model* or dynamic model* or aggregate model* or agent-based model* or differential equation* or monte carlo method* or microsimulation model* ordinary differential or complexity analys* or system? medicine or system? oriented approach* or decision support technique?).tw,kw,kf. |
| 7 | or/5-6 |
| 8 | and/4,7 |
| 9 | limit 8 to (English or French) |
| RESULTS: **253 STUDIES** | |

**Table S3. Search strategy and results for PsychINFO from inception to July 13, 2023**

| **#** | **SEARCH TERMS** |
| --- | --- |
| 1 | exp suicide |
| 2 | suicid*.ab |
| 3 | suicid*.ti |
| 4 | or/1-3 |
| 5 | exp *Systems Analysis/ or *systems theory/ or *exp Models, Theoretical/ |
| 6 | (system? thinking or system? science or system? approach* or system? theor* or system? analys* or system? dynamic? or dynamic system? or compartmental model* or mathematical model* or mechanistic model* or dynamic model* or aggregate model* or agent-based model* or differential equation* or monte carlo method* or microsimulation model* ordinary differential or complexity analys* or system? medicine or system? oriented approach* or decision support technique?).tw |
| 7 | or/5-6 |
| 8 | and/4,7 |
| 9 | limit 8 to (English or French) |
| RESULTS: **188 STUDIES** | |

**Table S4. Search strategy and results for Scopus from inception to July 13, 2023**

| **#** | **SEARCH TERMS** |
| --- | --- |
| 1 | (TITLE-ABS-KEY ( "suicide*" OR "suicidal" ) **AND** TITLE-ABS-KEY ( "system? thinking" OR "system? science" OR "system? approach*" OR "system? theor*" OR "system? analys*" OR "system? dynamic?" OR "dynamic system?" OR "compartmental model*" OR "mathematical model*" OR " mechanistic model*" OR "dynamic model*" OR "aggregate model*" OR "agent-based model*" OR "differential equation*" OR "monte carlo*" OR "microsimulation model*" OR "ordinary differential" OR "complexity analys*" OR "system? medicine" OR "system? oriented approach*" OR "decision support technique?" ) **AND LANGUAGE** ( English OR French ) ) |
| RESULTS: **536 STUDIES** | |

**Table S5. Search strategy and results for Compendex from inception to July 13, 2023**

| **#** | **SEARCH TERMS** |
| --- | --- |
| 1 | (TITLE-ABS-KEY (suicid* ) **AND** TITLE-ABS-KEY ( "system? thinking" OR "system? science" OR "system? approach*" OR "system? theor*" OR "system? analys*" OR "system? dynamic?" OR "system dynamic? " OR "dynamic system?" OR "compartmental model*" OR "mathematical model*" OR " mechanistic model*" OR "dynamic model*" OR "aggregate model*" OR "agent-based model*" OR "differential equation*" OR "monte carlo*" OR "microsimulation model*" OR "ordinary differential" OR "complexity analys*" OR "system? medicine" OR "system? oriented approach*" OR "decision support technique?" ) |
| **RESULTS: 33 STUDIES** | |

**Table S6. Search strategy and results for ACM Digital Library from inception to July 13, 2023**

| **#** | **SEARCH TERMS** |
| --- | --- |
| 1 | (suicid*) AND (system? thinking or system? science or system? approach* OR system? theor* OR system? analys* OR system? dynamic? OR dynamic system? OR compartmental model* OR mathematical model* OR mechanistic model* OR dynamic model* OR aggregate model* OR agent-based model* OR differential equation* OR monte carlo method* OR microsimulation model* OR ordinary differential OR complexity analys* OR system? medicine OR system? oriented approach* OR decision support technique?) **in Title** |
| 2 | (suicid*) AND (system? thinking OR system? science OR system? approach* OR system? theor* OR system? analys* OR system? dynamic? OR dynamic system? OR compartmental model* OR mathematical model* OR mechanistic model* OR dynamic model* OR aggregate model* OR agent-based model* OR differential equation* OR monte carlo method* OR microsimulation model* OR ordinary differential OR complexity analys* OR system? medicine OR system? oriented approach* OR decision support technique?) **in Abstract** |
| **RESULTS: 132 STUDIES** | |

**Table S7. Search strategy and results for IEEE Xplore Digital Library from inception to July 13, 2023**

| **#** | **SEARCH TERMS** |
| --- | --- |
| 1 | suicide OR suicidal (ALL Metadata)  AND  "system science" OR "systems science" OR "system approach" OR "systems approach" OR "system approaches" OR "system theory" OR "systems theory" OR "system theories" OR "systems theories" OR "system analysis" OR "systems analysis" OR "system analyses" OR "systems analyses" OR "system dynamic" OR "systems dynamic" OR "system dynamics" OR "systems dynamics" OR "dynamic system" OR "dynamic systems" OR "compartmental model" OR "compartmental models" OR "mathematical model" OR "mathematical models" OR "mechanistic model" OR "mechanistic models" OR "dynamic model" OR "dynamic models" OR "aggregate model" OR "aggregate models" OR "agent-based" OR "differential equation" OR "differential equations" OR "monte carlo" OR "microsimulation model" OR "microsimulation models" OR "ordinary differential" OR "complexity analysis" OR "complexity analyses" OR "system medicine" OR "systems medicine" OR "system oriented approach" OR "systems oriented approach" OR "system oriented approaches" OR "systems oriented approaches" OR "decision support technique" OR "decision support techniques" (ALL Metadata) |
| **RESULTS: 29 STUDIES** | |

**Table S8. Search strategy and results for medRxiv from inception to July 13, 2023**

| **#** | **SEARCH TERMS** |
| --- | --- |
| 1 | “Suicide” and “model” in title/abstract |
| 2 | “Suicidal” and “model” in title/abstract |
| 3 | “Suicide” and “models” in title/abstract |
| 4 | “Suicidal” and “models” in title/abstract |
| 5 | “Suicide” and “dynamic” in title/abstract |
| 6 | “Suicidal” and “dynamic” in title/abstract |
| **RESULTS: 136 STUDIES** | |

**Table S9. Characteristics of suicidal behavior studies using dynamic models**

| AUTHOR, YEAR | RESEARCH QUESTION | TARGET POPULATION | TIME HORIZON AND  TIME STEP | SUICIDE-RELATED BEHAVIOR OUTPUTS | PARTICIP. APPROACH | PLATFORM |
| --- | --- | --- | --- | --- | --- | --- |
| SYSTEM DYNAMICS MODELS | |  |  |  |  |  |
| Atkinson, 2019 (1) | Investigate the optimal combination of improvements in mental health service capacity and resources for service re-engagement for suicide and attempted suicide prevention | Population of Greater Western Sydney, Australia | 10 years (2018-2028);  time step N/R | - Suicide attempt hospitalizations  - Suicide deaths | Yes | Stella Architect |
| Atkinson, 2020 (2) | Identify (1) the impact of locally prioritized interventions and (2) their interactions on self-harm hospitalizations and suicide deaths | Rural population of Western New South Wales, Australia | 10 years (2021-2031);  0.4375 days | - Suicide attempts  - Suicide deaths | Yes | Stella Architect |
| De la Poza, 2018 (3) | Estimate the number of individuals at zero risk, pre-risk, risk and high risk of suicide, and the expected number of suicide deaths in Spain | Population of Spain, ages 16 to 78 | 3.5 years (2015-2019); 6 months | - Numbers of individuals at pre-risk, risk, and high risk of suicide  - Suicide deaths | No | N/R |
| De la Poza, 2019 (4) | Estimate the number of hidden suicide cases in Greece and compare results to those in Spain | Population of Greece and Spain, ages 16 to 78 | 3.5 years (2015-2019); 6 months | - Numbers of individuals at pre-risk, risk, and high risk of suicide  - Suicide deaths | No | N/R |
| Degenhardt, 2019 (5) | Model the impact of opioid agonist treatment scale-up on suicide, overdose, HIV, HCV, and accidental injuries among people who inject drugs | People who inject and who used to inject drugs in Kiev (Ukraine); Perry County (USA); and Tehran (Iran) | 20 years (2020-2040); time step N/R | - Suicide deaths | No | N/R |
| Iorfino, 2021 (6) | Explore the impact of technology-enabled care coordination on mental health and suicide outcomes, in a typical context and a public health crisis resulting from the pandemic and economic recession | Population of North Coast New South Wales, Australia | 9 years (2021-2030); 0.4375 days | - Self-harm hospitalizations  - Suicide deaths | Yes | Stella Architect |
| Occhipinti, 2021a (7) | Identify (1) the impact of locally prioritized interventions, (2) the value of investments across social determinants of mental health, and (3) the best combination of strategies, to reduce suicidal behaviors | Population of North Coast New South Wales, Australia | 20 years (2021-2041);  0.4375 days | - Suicide attempts  - Suicide deaths | Yes | Stella Architect |
| Occhipinti, 2021b (8) | Evaluate (1) the impact of an optimal combination of interventions to reduce suicide and the impact of (2) aligned and (3) differing program priorities between the federally governed primary health network and the state governed local health districts on suicide outcomes | Population of Hunter New England and Central Coast New South Wales, Australia | 20 years (2021-2041);  0.4375 days | - Self-harm hospitalizations  - Suicide deaths | Yes | Stella Architect |
| Occhipinti, 2022 (9) | Explore whether different simulated trajectories of suicide would result in different advice to decision makers on the optimal strategy to mitigate the impacts of the pandemic on mental health | Population of Perth South Primary Health Network, Australia | 20 years (2021-2041);  0.4375 days | - Suicide deaths | Yes | Stella Architect |
| Page,  2017 (10) | Investigate the impact of a combination of suicide intervention strategies | Population of Australia | 10 years (2015–2025);  time step N/R | - Hospital-treated attempted suicide  - Suicide deaths | No | N/R |
| Page,  2018a (11) | Compare the impact of a psychosocial therapy intervention on the incidence of suicide using a traditional epidemiological approach and a dynamic modeling approach | Population of Australia | 10 years (2016-2026);  time step N/R | - Self-harm hospitalizations  - Suicide deaths | No | N/R |
| Page,  2018b (12) | (1) Conceptualize and operationalize a suicide prevention model of the Western Sydney population; and (2) identify the combination of services and activities associated with suicide prevention | Population of Western Sydney, Australia | 10 years (2018-2028);  time step N/R | - Suicide attempt hospitalizations  - Suicide deaths | Yes | Stella Architect |
| Skinner, 2021 (13) | (1) Determine how regional variation in population and health system characteristics modifies the effects of suicide prevention measures on local suicide rates; (2) estimate the impact of optimal regional planning on the effectiveness of suicide prevention programmes; (3) assess the capacity of suboptimal commissioning decisions to limit potential benefit of local programme development | Population of 10 catchment areas of New South Wales, Australia | 10 years (2021-2031);  0.875 days | - Self-harm hospitalizations  - Suicide deaths | Yes | N/R |
| Vacher, 2022 (14) | Investigate the best combinations of health, economic, and social initiatives to improve post-pandemic mental health outcomes | Population of Victoria state, Australia | 6 years (2020-2026);  time step N/R | - Self-harm hospitalizations  - Suicide deaths | No | Stella Architect |
| Vacher, 2023 (15) | Simulate the impact of increasing direct access to mental health care professionals and increasing annual growth rate of mental health care capacity on population mental health indicators. | Population of New South Wales, Australia | 7 years (2021-2028); time step N/R | - Self-harm hospitalizations  - Suicide deaths | No | Stella Architect |
| AGENT-BASED MODELS | |  |  |  |  |  |
| Andarlia, 2021 (16) | Model the contagion effect of depression and the impact of repetitive transcranial magnetic stimulation interventions on depression and suicide | N/R | 5 years (1826 days);  1 day | - Suicide deaths | No | REPAST for Java and Eclipse |
| Cerdá,  2022 (17) | Estimate the change in rate of firearm homicide and suicide after disqualifying people from owning a firearm given prior alcohol- and drug-related misdemeanors | Population of New York City, ages 18 to 84 in 2000 | 30 years (2000-2030);  1 year | - Firearm-related suicide deaths | No | REPAST for Java and Eclipse |
| Keyes,  2019 (18) | Estimate the number of lives saved from firearm suicide with expansions of gun restrictions based on mental health compared with the number who would be unnecessarily restricted | Population of New York City, ages 18 to 84 in 2000 | 30 years (2000-2030);  1 year | - Firearm-related suicide deaths | No | AnyLogic |
| Liu,  2017 (19) | Investigate how self-evaluation and interpersonal loss influences suicide ideation | N/R | N/R | - Suicide deaths | No | N/R |
| Mesoudi, 2009 (20) | Explore if different social learning dynamics generate different spatial and temporal clusters of suicide | N/R | 100 generations;  time step not reported | - Suicide clustering | No | Netlogo |
| Morabito, 2015 (21) | Estimate the effect of social influence on suicide rates | N/R | 10 years (120 months);  1 month | - Suicide deaths | No | Borland C++ |
| MICROSIMULATION MODELS | |  |  |  |  |  |
| Zhang,  2023 (22) | Study the impact of different durations of antidepressant treatment on suicide risk among children and adolescents with major depressive disorder | Population of the United States, ages 10 to 17 | 1 year (52 weeks); 1 week | - Suicide rate  - Risk of suicide attempt | No | REPAST Simphony |

N/R: not reported; Particip. approach: participatory approach; REPAST: Recursive Porous Agent Simulation Toolkit

**Table S10. Summary of findings, limitations, and future research directions in suicidal behavior studies using dynamic models**

| AUTHOR, YEAR | RESEARCH QUESTION | MAIN FINDINGS | LIMITATIONS REPORTED BY THE AUTHORS | FUTURE RESEARCH DIRECTIONS REPORTED BY AUTHORS |
| --- | --- | --- | --- | --- |
| SYSTEM DYNAMICS MODELS | |  |  |  |
| Atkinson, 2019 (1) | Investigate the optimal combination of improvements in mental health service capacity and resources for service re-engagement for suicide and attempted suicide prevention | (1) A combination of increases in (i) hospital staffing, (ii) non-secondary health service capacity, (iii) mental health assessment capacity and (iv) service re-engagement had the greatest impact for reducing suicide and suicide attempts.  (2) Reduction in tertiary care would not adversely affect suicide behaviors if also accompanied by these increases. | (1) Model does not consider individual mechanisms and trajectories that affect outcomes  (2) Measurement bias in secondary data sources  (3) Under-enumeration of suicide cases  (4) Represents a limited set of interventions | (1) Development of enhanced primary care models (e.g., health-care homes, youth specific services) may be additional interventions for those at high risk but who do not need ongoing specialised services.  (2) Integration of costs into the model to facilitate health economic analyses and an exploration of barriers to implementation of selected interventions. |
| Atkinson, 2020 (2) | Identify (1) the impact of locally prioritized interventions and (2) their interactions on self-harm hospitalizations and suicide deaths | (1) Post-suicide attempt assertive aftercare was the most effective intervention for reducing suicide, followed by community support programmes.  (2) Some scenarios produced significantly lower or unintuitive impacts on suicide prevention. | (1) Measurement bias in secondary data sources  (2) Under-enumeration of suicide cases  (3) Hospitalization of suicide attempts only captures serious cases | Dynamic modeling can bring together the many pieces of a complex puzzle in a given context and provide new insights for decision makers and stakeholders. |
| De la Poza, 2018 (3) | Estimate the number of individuals at zero risk, pre-risk, risk and high risk of suicide, and the expected number of suicide deaths in Spain | The number of Spanish adults with zero and pre-risk of suicide is likely to decrease over time, while the number at risk and high-risk is likely to grow. | None specified | Model can be extrapolated and adapted to other regions where data are available. |
| De la Poza, 2019 (4) | Estimate the number of hidden suicide cases in Greece and compare results to those in Spain | (1) The number of Greek adults with zero-risk and pre-risk of suicide is likely to decrease over time, while the number at risk and high-risk is likely to grow.  (2) The number of people with at-risk and high-risk of suicide is likely to grow faster in Greece than in Spain, which may be partly explained by greater civilian firearm possession in Greece | None specified | Model can be extrapolated and adapted to other regions where data are available. |
| Degenhardt, 2019 (5) | Model the impact of opioid agonist treatment scale-up on suicide, overdose, HIV, HCV, and accidental injuries among people who inject drugs | (1) Increasing OAT coverage, improving retention, and providing OAT in prison would decrease deaths by suicide across all three settings (Kentucky (USA), Kiev (Ukraine), Tehran (Iran)).  (2) The reduction in suicide mortality would vary across settings. | Analyses focused on the impact of OAT on mortality rather than quality of life which will likely underestimate the population-level impact of OAT | Given that prevention of harm should be the goal of clinical guidance and strategies, dynamic models can provide insights to optimize combinations of pharmacological, psychological, and harm reduction interventions. |
| Iorfino, 2021 (6) | Explore the impact of technology-enabled care coordination on mental health and suicide outcomes, in a typical context and a public health crisis resulting from the pandemic and economic recession | The use of technology-enabled care coordination is likely to improve suicide outcomes in both a typical context and public health crisis context. | (1) Bias in secondary data sources  (2) Under-enumeration of suicide cases  (3) Uncertain generalizability to other regions | (1) Future work should also focus on validating the model outputs over time using local monitoring and evaluation of the implemented technology.  (2) Comparison of the modeled impacts applied to other regions may provide additional broader insights. |
| Occhipinti, 2021a (7) | Identify (1) the impact of locally prioritized interventions, (2) the value of investments across social determinants of mental health, and (3) the best combination of strategies, to reduce suicidal behaviors | (1) Improving social connectedness was the most effective intervention for reducing suicidal behavior.  (2) Reducing childhood adversity and increasing youth employment were the social determinant policies with the best impacts for youth population.  (3) A combination of social connectedness programs, technology-enabled coordinated care, post-attempt assertive aftercare, reductions in childhood adversity, and increasing youth employment had the best outcome, particularly in youth. | (1) Bias in secondary data sources  (2) Under-enumeration of suicide attempts and cases  (3) Uncertain generalizability to other regions | Dynamic models can provide regional decision makers and stakeholders the capacity to investigate alternative scenarios related to the timing of implementation of interventions, their scale and intensity, and to test alternative assumptions. |
| Occhipinti, 2021b (8) | Evaluate (1) the impact of an optimal combination of interventions to reduce suicide and the impact of (2) aligned and (3) differing program priorities between the federally governed primary health network and the state governed local health districts (LHD) on suicide outcomes | (1) Optimal impact of interventions can be achieved through independent decision making at the federal and state level.  (2) The combination of interventions to minimize suicide deaths differed between the two LHDs, but the difference in the number of suicides prevented was negligible.  (3) Competing priorities between the federal and state levels show a marked trade‑off between minimising suicide deaths versus minimising service disengagement. | (1) Bias in secondary data sources  (2) Under-enumeration of suicide attempts and cases  (3) Uncertain generalizability to other regions | (1) Systems modeling can provide regional decision analysis infrastructure to facilitate optimally coordinated federal and state investments.  (2) Competing priorities between the federal and state levels—each seeking to optimize the different outcomes they are responsible for—can affect the optimal impact of investments for suicide prevention. |
| Occhipinti, 2022 (9) | Explore whether different simulated trajectories of suicide would result in different advice to decision makers on the optimal strategy to mitigate the impacts of the pandemic on mental health | (1) The best intervention combination included post-suicide attempt care, community support programs to increase community connectedness, technology enabled care coordination, and family education and support.  (2) The best performing combination of three interventions were consistent across alternative COVID-19 mental health trajectories. | (1) Bias in secondary data sources  (2) Under-enumeration of suicide cases  (3) Uncertain generalizability to other regions | (1) The time horizon in dynamic models must be sufficiently long to capture the full effects of interventions.  (2) Need for more timely tracking and access to key population mental health indicators to refine the model over time and reduce uncertainty in mental health policy and planning decisions (3) Known sources of uncertainty should be acknowledged, and further research should focus on improving methods to measure model uncertainty. |
| Page,  2017 (10) | Investigate the impact of a combination of suicide intervention strategies | General practitioner training in suicide risk identification and post-suicide coordinated aftercare prevented the largest proportion of suicides | (1) Limited set of interventions  (2) Under-enumeration of suicide attempts and cases  (3) Does not consider individual mechanisms and trajectories  (3) Uncertain generalizability to other contexts | (1) Dynamic models can be calibrated for any given geographic context or subpopulation of interest, (2) and strengthened through participatory model-building exercises involving local stakeholders |
| Page,  2018a (11) | Compare the impact of a psychosocial therapy intervention on the incidence of suicide using a traditional epidemiological approach and a dynamic modeling approach | The psychosocial therapy intervention reduces the estimated number of suicides and suicide attempts but is insufficient to effectively reduce the ongoing increases in both outcomes. | Study was a simplified illustration of the use of dynamic modeling | Analytical models are needed that capture the dynamic and complex nature of the populations being targeted for suicide prevention, such as changes in population size, sociodemographic composition, psychological distress, and  differing uptake of interventions and services. |
| Page,  2018b (12) | (1) Build a suicide prevention model of the Western Sydney population and (2) identify the combination of services and activities associated with suicide prevention | (1) The largest number of cases of attempted suicide and suicide could be averted by focussing on assertive post-suicide aftercare, preventing disengagement, and reducing psychological distress and enhancing social connectedness in the general community.  (2) Interventions that identify those at suicide risk (e.g., GP gatekeeper training) showed little impact, unless there was also an increase in mental health service capacity. | (1) Does not consider individual mechanisms and trajectories  (2) Bias in secondary data sources  (3) Under-enumeration of suicide attempts and cases  (4) Limited set of interventions | The study demonstrated the utility of dynamic simulation models, co-designed with stakeholders to identify potential points for intervention activity. |
| Skinner, 2021 (13) | (1) Estimate the impact of optimal regional vs state-level planning on the effectiveness of suicide prevention programmes and (2) assess the capacity of suboptimal commissioning decisions to limit potential benefit of local programme development | (1) The most effective state-level combinations of four and five interventions prevented, respectively, 20% and 23% of suicide deaths. (2) Projected numbers of suicides under the optimal intervention scenarios at the regional level were up to 6% lower than corresponding numbers of suicides projected for the optimal state-level intervention combinations. | (1) Uncertain generalizability to other contexts  (2) Benefits of independent local planning are not considered  (3) Effect of the COVID-19 pandemic is not considered | Dynamic simulation models, co-developed using participatory methods, can help with the challenge of developing regional suicide prevention programmes while also accommodating heterogeneity in local services and community needs. |
| Vacher, 2022 (14) | Investigate the best combinations of health, economic, and social initiatives to improve post-pandemic mental health outcomes | (1) Assertive post-suicide attempt care is the most effective single intervention and  (2) financially supporting post-secondary students, employment programs, increasing mental health services capacities, technology-enabled care, and post-suicide attempt care are the most effective intervention combination.  (3) Population-wide community awareness campaigns are projected to worsen mental health outcomes when implemented on their own. | (1) Bias in secondary data sources  (2) Under-enumeration of suicide attempts and cases | Systems modeling is an important decision tool for testing scenarios to inform the  optimal mental health outcomes. |
| Vacher, 2023 (15) | Simulate the impact of increasing direct access to mental health care professionals and increasing annual growth rate of mental health care capacity on population mental health indicators. | (1) Increasing direct access proportion alone would increases self-harm hospitalizations in self-harm and deaths by and suicide due to increased waiting times  (2) Increasing annual rate of growth of mental health service capacity alone would reduce adverse outcomes.  (3) Combining increased direct access proportion with increased growth of mental health service capacity would provide greater gains than increasing service capacity alone. | (1) Data sources for model inputs may vary in quality  (2) Parameters values not available were estimated using constrained optimization and local verification | None specified |
| AGENT-BASED MODELS | |  |  |  |
| Andarlia, 2021 (16) | Model the contagion effect of depression and the impact of repetitive transcranial magnetic stimulation (rTMS) interventions on depression and suicide | (1) Greater contact rates increase the severity of depressive episodes and suicide deaths over time.  (2) Greater probability of receiving rTMS therapy reduces the number of depression cases and suicide deaths over time, in part by mitigating the contagion effect. | None specified | None specified |
| Cerdá,  2022 (17) | Estimate the change in rate of firearm homicide and suicide after disqualifying people from owning a firearm given prior alcohol- and drug-related misdemeanors | (1) Denying firearm access based on a history of drug/alcohol misdemeanors may produce a small reduction in firearm suicide rates in the population but a sizable reduction in high-risk groups meeting the denial criteria.  (2) The greatest reductions in firearm suicide are found among people with a prior history of alcohol misdemeanors. | (1) Validity of results depends on quality of calibration data  (2) Uncertain generalizability  (3) Model assumes that those meeting disqualification criteria could be identified and disarmed  (4) Modeled illegal firearms but empirical data on illegal firearm market missing | Future research will have to test whether findings generalize to other contexts |
| Keyes,  2019 (18) | Estimate the number of lives saved from firearm suicide with expansions of gun restrictions based on mental health compared with the number who would be unnecessarily restricted | (1) Firearm disqualifications based on psychiatric hospitalizations had no significant influence on population rates of suicide but decreased rates among those with psychiatric hospitalizations and with any past-year mental health treatment.  (2) Disqualification of anyone with mental health treatment significantly decreased suicide firearm rate | (1) Data for calibration does not reflect all of those who are disqualified from gun ownership  (2) Uncertain generalizability to areas outside New York  (3) Modeling assumptions may be wrong  (4) Limited quality of some parameters  (5) Does not include social network protective factors | (1) Further simulation modeling is needed to assess the emergence and exacerbation of disparities in racial/ethnic minorities.  (2) Should consider ownership disqualifications beyond mental health. |
| Liu,  2017 (19) | Investigate how self-evaluation and interpersonal loss influences suicide ideation | (1) Network structures and dynamics both influence suicide.  (2) Specifically, sparse networks and high clustering tend to have high suicide ratio relative to total deaths | None specified | (1) Further analyses on links between clustering, density, and degree distribution as well as influence of vulnerability and interpersonal loss.  (2) Enrichment of the model with more behavioral dimensions, such as agents establishing new ties following interpersonal loss dimension  (3) Model application of data-driven technologies (such as social media mining) for mental health intervention.  (4) Explore a similar model for other mental health outcomes. |
| Mesoudi, 2009 (20) | Explore if different social learning dynamics generate different spatial and temporal clusters of suicide | (1) Social learning within groups of agents generates spatiotemporal suicide clusters (2) Homophily generates clusters only when there is high individual variation in agents' suicide risk (3) Prestige and similarity bias were neither necessary nor sufficient for mass clusters. One-to-many transmissions was necessary but not sufficient for mass clusters. The three in combination generate mass clusters. | (1) Some model assumptions were simplifications  (2) No consideration of the social influence mechanism  (3) Mismatch between parameter values and equivalent real-life estimates | Results from the study can guide future empirical work by identifying the kinds of variables that may be important and that future empirical work should focus on. |
| Morabito, 2015 (21) | Estimate the effect of social influence on suicide rates | (1) Both greater density of agents and the presence of celebrity suicides increased suicide rates  (2) Both factors combined had a multiplicative effect on suicide rates | None specified | Future models could  (1) include local celebrity suicide  (2) increase number of suicide risk and protective factors  (3) vary these factors by regions |
| MICROSIMULATION MODELS | |  |  |  |
| Zhang,  2023 (22) | Study impact of different durations of antidepressant treatment on suicide risk among children and adolescents with major depressive disorder | Compared with receiving  no treatment, suicide rate and risk of suicide attempt both decreased with increasing duration of antidepressant  treatment | (1) Model did not account for all factors related to suicide  (2) Agent and environment interactions not considered  (3) Simulation does not reflect actual number of suicide events observed in real-world settings  (4) Potential biases from using multiple data sources | Future research could utilize agent-based modeling to account for agent-agent and agent-environment interactions |

**Table S11. Quality of reporting of system dynamics studies using the STRESS-SD guidelines**

| First author  Publication year | Atkinson 2019 (1) | Atkinson 2020 (2) | De la Poza 2018 (3) | De la Poza 2019 (4) | Degenhardt 2019 (5) | Iorfino 2021 (6) | Occhipinti 2021a (7) | Occhipinti 2021b (8) | Occhipinti 2022 (9) | Page 2017 (10) | Page 2018a (11) | Page 2018b (12) | Skinner 2021 (13) | Vacher 2022 (14) | Vacher 2023 (15) |
| --- | --- | --- | --- | --- | --- | --- | --- | --- | --- | --- | --- | --- | --- | --- | --- |
| RECOMMENDATION | **Met**  **(Yes/No)** | **Met**  **(Yes/No)** | **Met**  **(Yes/No)** | **Met**  **(Yes/No)** | **Met**  **(Yes/No)** | **Met**  **(Yes/No)** | **Met**  **(Yes/No)** | **Met**  **(Yes/No)** | **Met**  **(Yes/No)** | **Met**  **(Yes/No)** | **Met**  **(Yes/No)** | **Met**  **(Yes/No)** | **Met**  **(Yes/No)** | **Met**  **(Yes/No)** | **Met**  **(Yes/No)** |
| 1. OBJECTIVES |  |  |  |  |  |  |  |  |  |  |  |  |  |  |  |
| 1.1 Explains the purpose of the model | Y | Y | Y | Y | Y | Y | Y | Y | Y | Y | Y | Y | Y | Y | Y |
| 1.2 Describe all model outputs | Y | Y | Y | Y | Y | Y | Y | Y | Y | Y | N | Y | Y | Y | Y |
| 1.3 Describes experimentation aims | Y | Y | N/A | N/A | Y | Y | Y | Y | Y | Y | Y | Y | Y | Y | Y |
| 2. LOGIC |  |  |  |  |  |  |  |  |  |  |  |  |  |  |  |
| 2.1 Provides base model overview diagram | N | Y | Y | Y | N | Y | Y | Y | Y | Y | Y | Y | Y | Y | Y |
| 2.2 Provides base model logic | Y | Y | Y | Y | Y | Y | Y | Y | Y | Y | Y | Y | Y | Y | Y |
| 2.3 Provides scenario logic | Y | Y | N/A | N/A | Y | Y | Y | Y | Y | Y | Y | Y | Y | Y | Y |
| 2.4 Details of any algorithms in model | N | Y | Y | Y | Y | Y | Y | Y | Y | N | N | N | Y | N | Y |
| 2.5 Components |  |  |  |  |  |  |  |  |  |  |  |  |  |  |  |
| 2.5.1 Details of stocks/levels | N | Y | Y | Y | N | Y | Y | Y | Y | N | Y | N | Y | N | N |
| 2.5.2 Details of flows or rates | N | Y | Y | Y | N | Y | Y | Y | Y | N | N | N | Y | N | N |
| 2.5.3 Details of constants/converter/  auxiliaries | N | Y | Y | Y | N | Y | Y | Y | Y | N | N | N | Y | N | N |
| 2.5.4 Details of graphical functions/ lookup tables | N/A | N/A | N/A | N/A | N/A | N/A | N/A | N/A | N/A | N/A | N/A | N/A | N/A | N/A | N/A |
| 2.5.5 Details of sources/sinks | N | Y | N | N | N | Y | Y | Y | Y | Y | Y | N | Y | N | N |
| 3. DATA |  |  |  |  |  |  |  |  |  |  |  |  |  |  |  |
| 3.1 Lists and details all data sources | Y | Y | Y | Y | Y | Y | Y | Y | Y | N | N | Y | Y | Y | Y |
| 3.2 Details of pre-processing data manipulation | N/A | N/A | N/A | N/A | N/A | N/A | N/A | N/A | N/A | N/A | N/A | N/A | N/A | N/A | N/A |
| 3.3 Details of input parameters | N | Y | Y | Y | Y | Y | Y | Y | Y | N | N | Y | Y | Y | Y |
| 3.4 Details of assumptions | Y | Y | Y | Y | Y | Y | Y | Y | Y | N | N | Y | Y | Y | Y |
| 4. EXPERIMENTATION |  |  |  |  |  |  |  |  |  |  |  |  |  |  |  |
| 4.1 Details of initialization | N | Y | Y | Y | Y | Y | Y | Y | Y | N | N | Y | Y | N | N |
| 4.2 Describes run length and time units | **N** | Y | Y | Y | N | Y | Y | Y | Y | N | N | N | Y | N | N |
| 4.3 Reports the estimation approach | Y | Y | N/A | N/A | Y | Y | Y | Y | Y | N | N | Y | Y | Y | Y |
| 5. IMPLEMENTATION |  |  |  |  |  |  |  |  |  |  |  |  |  |  |  |
| 5.1 States the software or programming language used | Y | Y | N | N | N | Y | Y | Y | Y | N | N | Y | Y | Y | Y |
| 5.2 *For stochastic models only,* states algorithm used to generate random samples | N/A | N/A | N/A | N/A | N/A | N/A | N/A | N/A | N/A | N/A | N/A | N/A | N/A | N/A | N/A |
| 5.3 Reports the model execution/integration method | N | N | N | N | N | N | N | N | N | N | N | N | N | N | N |
| 5.4 States the system specification | N | N | N | N | N | N | N | N | N | N | N | N | N | N | N |
| 6. CODE ACCESS |  |  |  |  |  |  |  |  |  |  |  |  |  |  |  |
| 6.1 Provides a computer model sharing statement | N | N | N | N | N | N | N | N | N | N | N | N | N | N | N |

Y: Yes; N: No; N/A: not applicable

**Table S12. Quality of reporting of agent-based modeling studies using the STRESS-ABM guidelines**

| First author  Publication year | Andarlia 2021 (16) | Cerdá  2022 (17) | Keyes  2019 (18) | Liu  2017 (19) | Mesoudi  2009 (20) | Morabito  2015 (21) |
| --- | --- | --- | --- | --- | --- | --- |
| RECOMMENDATION | **Met**  **(Yes/No)** | **Met**  **(Yes/No)** | **Met**  **(Yes/No)** | **Met**  **(Yes/No)** | **Met**  **(Yes/No)** | **Met (Yes/No)** |
| 1. OBJECTIVES |  |  |  |  |  |  |
| 1.1 Explains the purpose of the model | Y | Y | Y | Y | Y | Y |
| 1.2 Describe all model outputs | N | Y | Y | N | Y | Y |
| 1.3 Describes experimentation aims | N | Y | Y | Y | Y | Y |
| 2. LOGIC |  |  |  |  |  |  |
| 2.1 Provides base model overview diagram | Y | Y | Y | N | N | N |
| 2.2 Provides base model logic | Y | Y | Y | Y | Y | Y |
| 2.3 Provides scenario logic | Y | Y | Y | Y | Y | Y |
| 2.4 Details of any algorithms in model | N | Y | Y | Y | N | Y |
| 2.5 Components |  |  |  |  |  |  |
| 2.5.1 Details of environment | Y | Y | Y | Y | N | Y |
| 2.5.2 Details of agents | Y | Y | Y | Y | Y | Y |
| 2.5.3 Details of interaction topology | N/A | Y | Y | N/A | N/A | N/A |
| 2.5.4 Details of entry/exit | Y | Y | Y | N | N | N |
| 3. DATA |  |  |  |  |  |  |
| 3.1 Lists and details all data sources | Y | Y | Y | N | N | Y |
| 3.2 Details of pre-processing data manipulation | N/A | N/A | N/A | N/A | N/A | N/A |
| 3.3 Details of input parameters | Y | Y | Y | Y | Y | Y |
| 3.4 Details of assumptions | Y | Y | Y | Y | Y | Y |
| 4. EXPERIMENTATION |  |  |  |  |  |  |
| 4.1 Details of initialisation | N | Y | Y | N | Y | Y |
| 4.2 Describes run length and time units | Y | Y | Y | N | Y | Y |
| 4.3 Reports the estimation approach | N | Y | Y | N | Y | Y |
| 5. IMPLEMENTATION |  |  |  |  |  |  |
| 5.1 States the software or programming language used | Y | Y | Y | N | Y | Y |
| 5.2 *For stochastic models only,* states algorithm used to generate random samples | N/A | N/A | N/A | N | N | N |
| 5.3 Reports the model execution/ integration method | Y | Y | Y | N | Y | Y |
| 5.4 States the system specification | N | N | N | N | N | N |
| 6. CODE ACCESS |  |  |  |  |  |  |
| 6.1 Provides a computer model sharing statement | N | N | N | N | Y | N |

Y: Yes; N: No; N/A: not applicable

**Table S13. Quality of reporting of microsimulation studies using the STRESS-ABM guidelines**

| First author  Publication year | Zhang  2023 |
| --- | --- |
| RECOMMENDATION | **Met (Yes/No)** |
| 1. OBJECTIVES |  |
| 1.1 Explains the purpose of the model | Y |
| 1.2 Describe all model outputs | Y |
| 1.3 Describes experimentation aims | Y |
| 2. LOGIC |  |
| 2.1 Provides base model overview diagram | Y |
| 2.2 Provides base model logic | Y |
| 2.3 Provides scenario logic | Y |
| 2.4 Details of any algorithms in model | Y |
| 2.5 Components |  |
| 2.5.1 Details of environment | N/A |
| 2.5.2 Details of agents | Y |
| 2.5.3 Details of interaction topology | N/A |
| 2.5.4 Details of entry/exit | N/A |
| 3. DATA |  |
| 3.1 Lists and details all data sources | Y |
| 3.2 Details of pre-processing data manipulation | N/A |
| 3.3 Details of input parameters | Y |
| 3.4 Details of assumptions | Y |
| 4. EXPERIMENTATION |  |
| 4.1 Details of initialisation | N/A |
| 4.2 Describes run length and time units | Y |
| 4.3 Reports the estimation approach | Y |
| 5. IMPLEMENTATION |  |
| 5.1 States the software or programming language used | Y |
| 5.2 *For stochastic models only,* states algorithm used to generate random samples | N/A |
| 5.3 Reports the model execution/ integration method | Y |
| 5.4 States the system specification | N |
| 6. CODE ACCESS |  |
| 6.1 Provides a computer model sharing statement | N |

Y: Yes; N: No; N/A: not applicable

## **References**

1. Atkinson JA, Page A, Heffernan M, McDonnell G, Prodan A, Campos B, Meadows G, Hickie IB. The impact of strengthening mental health services to prevent suicidal behaviour. Aust N Z J Psychiatry. 2019;53(7):642-50. PMID: <https://www.ncbi.nlm.nih.gov/pubmed/30541332>. doi: <https://doi.org/https://dx.doi.org/10.1177/0004867418817381>. URI: <https://ovidsp.ovid.com/ovidweb.cgi?T=JS&CSC=Y&NEWS=N&PAGE=fulltext&D=med16&AN=30541332http://CT4XW3QJ9Z.search.serialssolutions.com/?url_ver=Z39.88-2004&rft_val_fmt=info:ofi/fmt:kev:mtx:journal&rfr_id=info:sid/Ovid:med16&rft.genre=article&rft_id=info:doi>.

2. Atkinson JA, Skinner A, Hackney S, Mason L, Heffernan M, Currier D, King K, Pirkis J. Systems modelling and simulation to inform strategic decision making for suicide prevention in rural New South Wales (Australia). Aust N Z J Psychiatry. 2020;54(9):892-901. PMID: <https://www.ncbi.nlm.nih.gov/pubmed/32551878>. doi: <https://doi.org/https://dx.doi.org/10.1177/0004867420932639>. URI: <https://ovidsp.ovid.com/ovidweb.cgi?T=JS&CSC=Y&NEWS=N&PAGE=fulltext&D=med17&AN=32551878http://CT4XW3QJ9Z.search.serialssolutions.com/?url_ver=Z39.88-2004&rft_val_fmt=info:ofi/fmt:kev:mtx:journal&rfr_id=info:sid/Ovid:med17&rft.genre=article&rft_id=info:doi>.

3. De la Poza E, Jodar L. A Short-Term Population Model of the Suicide Risk: The Case of Spain. Cult Med Psychiatry. 2018;42(4):800-20. PMID: <https://www.ncbi.nlm.nih.gov/pubmed/29948433>. doi: <https://doi.org/https://dx.doi.org/10.1007/s11013-018-9589-4>. URI: <https://ovidsp.ovid.com/ovidweb.cgi?T=JS&CSC=Y&NEWS=N&PAGE=fulltext&D=med15&AN=29948433http://CT4XW3QJ9Z.search.serialssolutions.com/?url_ver=Z39.88-2004&rft_val_fmt=info:ofi/fmt:kev:mtx:journal&rfr_id=info:sid/Ovid:med15&rft.genre=article&rft_id=info:doi>.

4. De la Poza E, Jódar L, Douklia G. Modeling the spread of suicide in Greece. Complex Syst. 2019;28(4):475-89. doi: <https://doi.org/10.25088/ComplexSystems.28.4.475>. URI: <https://www.scopus.com/inward/record.uri?eid=2-s2.0-85076905453&doi=10.25088%2fComplexSystems.28.4.475&partnerID=40&md5=90610c5ca15372b710c5f188073921c0>.

5. Degenhardt L, Grebely J, Stone J, Hickman M, Vickerman P, Marshall BDL, Bruneau J, Altice FL, Henderson G, Rahimi-Movaghar A, Larney S. Global patterns of opioid use and dependence: harms to populations, interventions, and future action. Lancet. 2019;394(10208):1560-79. PMID: <https://www.ncbi.nlm.nih.gov/pubmed/31657732>. doi: <https://doi.org/https://dx.doi.org/10.1016/S0140-6736(19)32229-9>. URI: <https://ovidsp.ovid.com/ovidweb.cgi?T=JS&CSC=Y&NEWS=N&PAGE=fulltext&D=med16&AN=31657732http://CT4XW3QJ9Z.search.serialssolutions.com/?url_ver=Z39.88-2004&rft_val_fmt=info:ofi/fmt:kev:mtx:journal&rfr_id=info:sid/Ovid:med16&rft.genre=article&rft_id=info:doi>.

6. Iorfino F, Occhipinti JA, Skinner A, Davenport T, Rowe S, Prodan A, Sturgess J, Hickie IB. The Impact of Technology-Enabled Care Coordination in a Complex Mental Health System: A Local System Dynamics Model. J Med Internet Res. 2021;23(6):e25331. PMID: <https://www.ncbi.nlm.nih.gov/pubmed/34077384>. doi: <https://doi.org/https://dx.doi.org/10.2196/25331>. URI: <https://ovidsp.ovid.com/ovidweb.cgi?T=JS&CSC=Y&NEWS=N&PAGE=fulltext&D=med19&AN=34077384http://CT4XW3QJ9Z.search.serialssolutions.com/?url_ver=Z39.88-2004&rft_val_fmt=info:ofi/fmt:kev:mtx:journal&rfr_id=info:sid/Ovid:med19&rft.genre=article&rft_id=info:doi>.

7. Occhipinti JA, Skinner A, Iorfino F, Lawson K, Sturgess J, Burgess W, Davenport T, Hudson D, Hickie I. Reducing youth suicide: systems modelling and simulation to guide targeted investments across the determinants. BMC Med. 2021;19(1):61. PMID: <https://www.ncbi.nlm.nih.gov/pubmed/34045644>. doi: <https://doi.org/https://dx.doi.org/10.1038/s41598-021-90762-x>. URI: <https://ovidsp.ovid.com/ovidweb.cgi?T=JS&CSC=Y&NEWS=N&PAGE=fulltext&D=med19&AN=34045644http://CT4XW3QJ9Z.search.serialssolutions.com/?url_ver=Z39.88-2004&rft_val_fmt=info:ofi/fmt:kev:mtx:journal&rfr_id=info:sid/Ovid:med19&rft.genre=article&rft_id=info:doi>.

8. Occhipinti JA, Skinner A, Carter S, Heath J, Lawson K, McGill K, McClure R, Hickie IB. Federal and state cooperation necessary but not sufficient for effective regional mental health systems: insights from systems modelling and simulation. Scientific Reports. 2021;11(1):1-12. PMID: <https://www.ncbi.nlm.nih.gov/pubmed/33706764>. doi: <https://doi.org/https://dx.doi.org/10.1186/s12916-021-01935-4>. URI: <https://ovidsp.ovid.com/ovidweb.cgi?T=JS&CSC=Y&NEWS=N&PAGE=fulltext&D=med19&AN=33706764http://CT4XW3QJ9Z.search.serialssolutions.com/?url_ver=Z39.88-2004&rft_val_fmt=info:ofi/fmt:kev:mtx:journal&rfr_id=info:sid/Ovid:med19&rft.genre=article&rft_id=info:doi>.

9. Occhipinti JA, Rose D, Skinner A, Rock D, Song YJC, Prodan A, Rosenberg S, Freebairn L, Vacher C, Hickie IB. Sound Decision Making in Uncertain Times: Can Systems Modelling Be Useful for Informing Policy and Planning for Suicide Prevention? Int J Environ Res Public Health. 2022;19(3):27. PMID: <https://www.ncbi.nlm.nih.gov/pubmed/35162491>. doi: <https://doi.org/https://dx.doi.org/10.1038/s41598-021-90762-x>. URI: <https://ovidsp.ovid.com/ovidweb.cgi?T=JS&CSC=Y&NEWS=N&PAGE=fulltext&D=med20&AN=35162491http://CT4XW3QJ9Z.search.serialssolutions.com/?url_ver=Z39.88-2004&rft_val_fmt=info:ofi/fmt:kev:mtx:journal&rfr_id=info:sid/Ovid:med20&rft.genre=article&rft_id=info:doi>.

10. Page A, Atkinson JA, Heffernan M, McDonnell G, Hickie I. A decision-support tool to inform Australian strategies for preventing suicide and suicidal behaviour. Public health res. 2017;27(2):27. PMID: <https://www.ncbi.nlm.nih.gov/pubmed/28474054>. doi: <https://doi.org/https://dx.doi.org/10.17061/phrp2721717>. URI: <https://ovidsp.ovid.com/ovidweb.cgi?T=JS&CSC=Y&NEWS=N&PAGE=fulltext&D=med14&AN=28474054http://CT4XW3QJ9Z.search.serialssolutions.com/?url_ver=Z39.88-2004&rft_val_fmt=info:ofi/fmt:kev:mtx:journal&rfr_id=info:sid/Ovid:med14&rft.genre=article&rft_id=info:doi>.

11. Page A, Atkinson JA, Heffernan M, McDonnell G, Prodan A, Osgood N, Hickie I. Static metrics of impact for a dynamic problem: The need for smarter tools to guide suicide prevention planning and investment. Aust N Z J Psychiatry. 2018;52(7):660-7. PMID: <https://www.ncbi.nlm.nih.gov/pubmed/29359569>. doi: <https://doi.org/https://dx.doi.org/10.1177/0004867417752866>. URI: <https://ovidsp.ovid.com/ovidweb.cgi?T=JS&CSC=Y&NEWS=N&PAGE=fulltext&D=med15&AN=29359569http://CT4XW3QJ9Z.search.serialssolutions.com/?url_ver=Z39.88-2004&rft_val_fmt=info:ofi/fmt:kev:mtx:journal&rfr_id=info:sid/Ovid:med15&rft.genre=article&rft_id=info:doi>.

12. Page A, Atkinson JA, Campos W, Heffernan M, Ferdousi S, Power A, McDonnell G, Maranan N, Hickie I. A decision support tool to inform local suicide prevention activity in Greater Western Sydney (Australia). Aust N Z J Psychiatry. 2018;52(10):983-93. PMID: <https://www.ncbi.nlm.nih.gov/pubmed/29671335>. doi: <https://doi.org/https://dx.doi.org/10.1177/0004867418767315>. URI: <https://ovidsp.ovid.com/ovidweb.cgi?T=JS&CSC=Y&NEWS=N&PAGE=fulltext&D=med15&AN=29671335http://CT4XW3QJ9Z.search.serialssolutions.com/?url_ver=Z39.88-2004&rft_val_fmt=info:ofi/fmt:kev:mtx:journal&rfr_id=info:sid/Ovid:med15&rft.genre=article&rft_id=info:doi>.

13. Skinner A, Occhipinti JA, Song YJC, Hickie IB. Regional suicide prevention planning: A dynamic simulation modelling analysis. BJPsych Open. 2021;7(5) (no pagination). PMID: <https://www.ncbi.nlm.nih.gov/pubmed/2017510356>. doi: <https://doi.org/https://dx.doi.org/10.1192/bjo.2021.989>. URI: <https://www.cambridge.org/core/journals/bjpsych-openhttps://ovidsp.ovid.com/ovidweb.cgi?T=JS&CSC=Y&NEWS=N&PAGE=fulltext&D=emexb&AN=2017510356http://CT4XW3QJ9Z.search.serialssolutions.com/?url_ver=Z39.88-2004&rft_val_fmt=info:ofi/fmt:kev:mtx:journal&rfr_id>.

14. Vacher C, Ho N, Skinner A, Robinson J, Freebairn L, Lee GY, Iorfino F, Prodan A, Song YJC, Occhipinti JA, Hickie IB. Optimizing Strategies for Improving Mental Health in Victoria, Australia during the COVID-19 Era: A System Dynamics Modelling Study. Int J Environ Res Public Health. 2022;19(11):26. PMID: <https://www.ncbi.nlm.nih.gov/pubmed/35682058>. doi: <https://doi.org/https://dx.doi.org/10.3390/ijerph19116470>. URI: <https://ovidsp.ovid.com/ovidweb.cgi?T=JS&CSC=Y&NEWS=N&PAGE=fulltext&D=medl&AN=35682058http://CT4XW3QJ9Z.search.serialssolutions.com/?url_ver=Z39.88-2004&rft_val_fmt=info:ofi/fmt:kev:mtx:journal&rfr_id=info:sid/Ovid:medl&rft.genre=article&rft_id=info:doi/1>.

15. Vacher C, Skinner A, Occhipinti J-A, Rosenberg S, Ho N, Song YJC, Hickie IB. Improving access to mental health care: a system dynamics model of direct access to specialist care and accelerated specialist service capacity growth. Medical Journal of Australia. 2023;218(7):309-14. doi: <https://doi.org/https://doi.org/10.5694/mja2.51903>. URI: <https://onlinelibrary.wiley.com/doi/abs/10.5694/mja2.51903>.

16. Andarlia HT, Gunawan I, editors. An Agent-Based Model of Contagion Effects in Affected Depression and Its Recovery Process2021: IOP Publishing Ltd.

17. Cerda M, Hamilton AD, Tracy M, Branas C, Fink D, Keyes KM. Would restricting firearm purchases due to alcohol- and drug-related misdemeanor offenses reduce firearm homicide and suicide? An agent-based simulation. Inj Epidemiol. 2022;9(1):17. PMID: <https://www.ncbi.nlm.nih.gov/pubmed/35681243>. doi: <https://doi.org/https://dx.doi.org/10.1186/s40621-022-00381-x>. URI: <https://ovidsp.ovid.com/ovidweb.cgi?T=JS&CSC=Y&NEWS=N&PAGE=fulltext&D=pmnm&AN=35681243http://CT4XW3QJ9Z.search.serialssolutions.com/?url_ver=Z39.88-2004&rft_val_fmt=info:ofi/fmt:kev:mtx:journal&rfr_id=info:sid/Ovid:pmnm&rft.genre=article&rft_id=info:doi/1>.

18. Keyes KM, Hamilton A, Swanson J, Tracy M, Cerda M. Simulating the Suicide Prevention Effects of Firearms Restrictions Based on Psychiatric Hospitalization and Treatment Records: Social Benefits and Unintended Adverse Consequences. Am J Public Health. 2019;109(S3):S236-S43. PMID: <https://www.ncbi.nlm.nih.gov/pubmed/31242005>. doi: <https://doi.org/https://dx.doi.org/10.2105/AJPH.2019.305041>. URI: <https://ovidsp.ovid.com/ovidweb.cgi?T=JS&CSC=Y&NEWS=N&PAGE=fulltext&D=med16&AN=31242005http://CT4XW3QJ9Z.search.serialssolutions.com/?url_ver=Z39.88-2004&rft_val_fmt=info:ofi/fmt:kev:mtx:journal&rfr_id=info:sid/Ovid:med16&rft.genre=article&rft_id=info:doi>.

19. Liu J, Li L, Russell K. What Becomes of the Broken Hearted? An Agent-Based Approach to Self-Evaluation, Interpersonal Loss, and Suicide Ideation. Aamas '17. 2017:436–45.

20. Mesoudi A. The cultural dynamics of copycat suicide. PLoS One. 2009;4(9):e7252. PMID: <https://www.ncbi.nlm.nih.gov/pubmed/19789643>. doi: <https://doi.org/10.1371/journal.pone.0007252>.

21. Morabito PN, Cook AV, Homan CM, Long ME. Agent-Based Models of Copycat Suicide. Springer Verlag; 2015. p. 369-74.

22. Zhang C, Zafari Z, Slejko JF, Castillo WC, Reeves GM, dosReis S. Impact of Undertreatment of Depression on Suicide Risk Among Children and Adolescents With Major Depressive Disorder: A Microsimulation Study. American Journal of Epidemiology. 2023;192(6):929-38. doi: <https://doi.org/10.1093/aje/kwad022>. URI: <https://doi.org/10.1093/aje/kwad022>.
